# Systematic monocyte subset analysis reveals differential contribution of Notch signaling components to monocyte heterogeneity

**DOI:** 10.1016/j.isci.2025.113795

**Published:** 2025-10-16

**Authors:** Yuangao Xu, Tamar Kapanadze, Svenja Gaedcke, Adan Chari Jirmo, Stefan Sablotny, Frauline Nicole Schroth, Susanne Hille, Oliver J. Müller, Matthias Lochner, Hermann Haller, Kai Schmidt-Ott, Jaba Gamrekelashvili, Florian P. Limbourg

**Affiliations:** 1 Vascular Medicine Research, Department of Nephrology and Hypertension, Hannover Medical School, 30625 Hannover, Germany; 2 Department of Nephrology and Hypertension, Hannover Medical School, 30625 Hannover, Germany; 3 Department of Respiratory Medicine and Infectious Diseases, Hannover Medical School, 30625 Hannover, Germany; 4 Biomedical Research in Endstage and Obstructive Lung Disease (BREATH), Member of the German Center for Lung Research (DZL), Hannover Medical School, 30625 Hannover, Germany; 5 Department of Pediatric Surgery, Hannover Medical School, 30625 Hannover, Germany; 6 MDI Biological Laboratory, Bar Harbor, ME, USA; 7 Department of Internal Medicine V, University Hospital Schleswig-Holstein, University of Kiel, 24105 Kiel, Germany; 8 Institute for Experimental Infection Research, TWINCORE, Centre for Experimental and Clinical Infection Research, a joint venture between the Helmholtz Centre for Infection Research Braunschweig and the Hannover Medical School, 30625 Hannover, Germany; 9 Institute of Medical Microbiology and Hospital Epidemiology, Hannover Medical School, 30625 Hannover, Germany

**Keywords:** molecular biology, Immunology, cell biolog

## Abstract

Monocyte heterogeneity and plasticity create a spectrum of phagocytes essential for innate immune functions, which is partially regulated by Notch signaling. Using systematic monocyte subset analysis in different compartments, we here confirm that monocyte heterogeneity extends beyond the Ly6C^hi^ and Ly6C^lo^ monocytes and is regulated by Notch. Employing different monocyte-lineage-development-specific Cre-deleter strains in combination with conditional alleles for the receptor Notch2 or the Notch nuclear mediator Rbpj, we also show that subset development is differentially regulated by Notch-signaling components. Deletion of Notch2 broadly affects development of Ly6C^lo^ monocytes, or related monocyte subsets, and alters monocyte phenotypes, while deletion of Rbpj has more restricted effects, mostly on monocyte phenotypes. Furthermore, the developmental plasticity of Ly6C^hi^ monocyte subsets *in vitro* is regulated by Notch2 but dependent on the context of specific Notch ligand and myeloid growth factor. Thus, Notch signaling components differentially regulate monocyte heterogeneity and plasticity.

## Highlights

• Notch2 controls monocyte conversion and phenotype and defines heterogeneity of monocyte pools
• Rbpj regulates monocyte phenotype and migration, contributing to monocyte heterogeneity
• DLL1/4-Notch2 controls conversion of DC/GC-like monocytes in a growth-factor-dependent manner
• DLL1/4-Notch2 regulates GC-like monocyte plasticity toward monocyte subsets and MoDCs

## Graphical abstract

**Figure:**
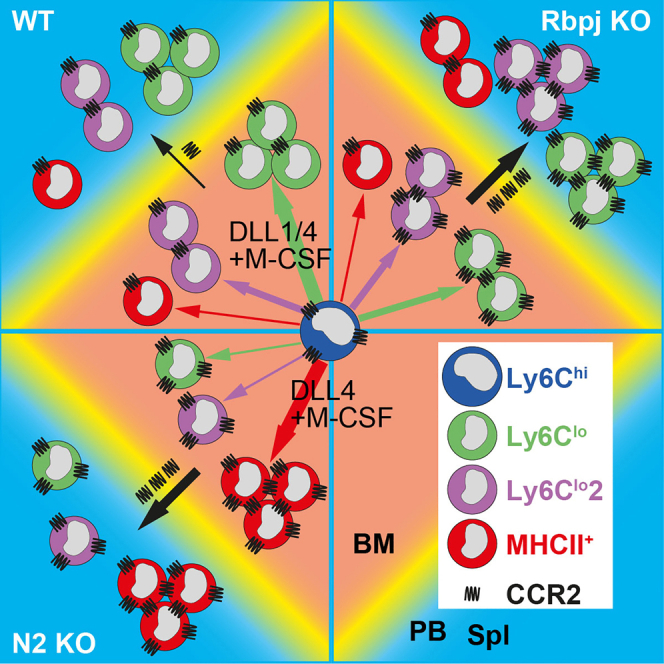

## Introduction

Monocytes, macrophages (MFs), and dendritic cells (DCs) are key constituents of the mononuclear phagocyte system (MPS) involved in inflammation and the immune response. Within monocytes, two major subsets are discriminated in mice and humans based on phenotypic and functional features. In mice, inflammatory, classical, or Ly6C^hi^ monocytes are the prevalent subtype with high plasticity. In contrast, Ly6C^lo^ or non-classical monocytes are less frequent but represent a distinct subset with blood-vessel-patrolling behavior, for which they also have been termed patrolling monocytes.^1^^,^^2^^,^^3^ A third population, intermediate monocytes, was also recently proposed to be Ly6C^int^ monocytes in mice expressing CD209a^4^ with still unknown origin and function.

Ly6C^hi^ monocytes are short-lived and develop from myeloid progenitors in the central compartment of the bone marrow.^5^^,^^6^^,^^7^^,^^8^ They circulate in the peripheral blood (PB) and emigrate to the spleen or target organs, where they differentiate into a spectrum of effector cells, such as MFs, monocyte-derived dendritic cells (MoDCs), or Ly6C^lo^ monocytes.^8^^,^^9^^,^^10^^,^^11^^,^^12^ Ly6C^lo^ monocytes, in contrast, are relatively long-lived and develop from Ly6C^hi^ monocytes in a regulated process termed “monocyte conversion.”^9^^,^^13^ Besides blood vessel monitoring, they are involved in antitumor immunity.^14^^,^^15^^,^^16^ Patrolling monocytes may have the potential to differentiate further into specialized macrophage subsets in the spleen or lung.^17^^,^^18^^,^^19^

Ly6C^hi^ monocytes possess intrinsic developmental plasticity, which ensures a rapid shift in developmental fate depending on environmental signals, including niche-specific factors, inflammation, or pathogens. Ly6C^hi^ monocytes are heterogeneous and consist of at least two subsets.^20^ Granulocyte (GC)-like monocytes develop via granulocyte-monocyte progenitors (GMPs) and common monocyte progenitors (cMoPs) and express genes characteristic of neutrophilic granulocytes (*Chil3, Prtn3, Elane*, and *Mpo*). Dendritic cell (DC)-like monocytes are thought to be derived from the monocyte-DC progenitors (MDPs) and express genes related to dendritic cells (*Cd74*, *H2-Ab1*, and *Cd209a*).^20^^,^^21^ Both GC-like and DC-like subsets exist in the steady state, differentiate into different tissue MFs in the lung, intestine, or brain,^22^ and expand in response to inflammatory stimulation with CpG or LPS, respectively.^20^^,^^21^ The heterogeneity of inflammatory monocytes further includes Ym1^+^ Ly6C^hi^ or Cxcl10 monocytes, which expand under pathologic conditions.^23^^,^^24^

Furthermore, heterogeneity also exists in the pool of Ly6C^lo^ monocytes. Prototypical Ly6C^lo^ monocytes are CD11c^lo^MHCII^neg^CD43^+^ (Ly6C^lo^) or CD11c^neg^MHCII^neg^CD43^+^ (Ly6C^lo^2) and develop from Ly6C^hi^ monocytes by monocyte conversion, regulated by Notch signaling.^13^^,^^25^ These Notch2-dependent Ly6C^lo^ monocytes also require *Nr4a1*, *Bcl6*, *Irf2*, and *Cebpb* for development and maintenance.^4^^,^^25^ There is also a Notch2-independent subset, which has been reported to develop from an unknown progenitor in the absence of Ly6C^hi^ monocytes.^26^ A third subset (CD11c^neg^MHCII^+^CD43^neg^) of Ly6C^lo^ monocytes (MHCII^+^) is a minor subset in steady-state conditions but expands in Notch2-deficient mice^13^ and in models of systemic inflammation.^27^

Notch is a master regulator of myeloid cell development. Monocyte conversion in steady-state conditions depends on monocyte Notch2 signaling, which is activated by the Notch ligand Delta-like 1 (Dll1) *in vivo* and *in vitro*. This signaling further interacts with the TLR-Myd88 pathway during inflammation.^13^^,^^27^ Direct nuclear, transcriptional effects of Notch signaling are mediated by Rbpj (recombination signal binding protein for immunoglobulin κJ region), which is required for canonical Notch signaling, but noncanonical effects of Notch signaling have also been described. Deletion of Rbpj in monocytes upregulates CCR2 expression and thus influences Ly6C^lo^ monocyte distribution.^19^ Notch-Rbpj signaling also controls monocyte-to-macrophage transition in liver disease and the development of protective Ly6C^lo^ monocytes.^28^^,^^29^ Similarly, Notch2 and Rbpj are required for the formation of conventional DCs.^30^^,^^31^^,^^32^

Employing a systematic monocyte subset characterization, we here confirm that monocyte heterogeneity extends beyond the previously appreciated subsets of Ly6C^hi^ and Ly6C^lo^ monocytes, which in part is compartment-specific and regulated by Notch. Using monocyte-lineage-development-specific Cre-deleter strains targeting conditional alleles for Notch2 or Rbpj, we also show that subset development is differentially affected by deficiency in Notch-signaling components and by specific growth factors.

## Results

### Compartment-specific differences in Notch signaling in monocyte subsets

**Figure 1:**
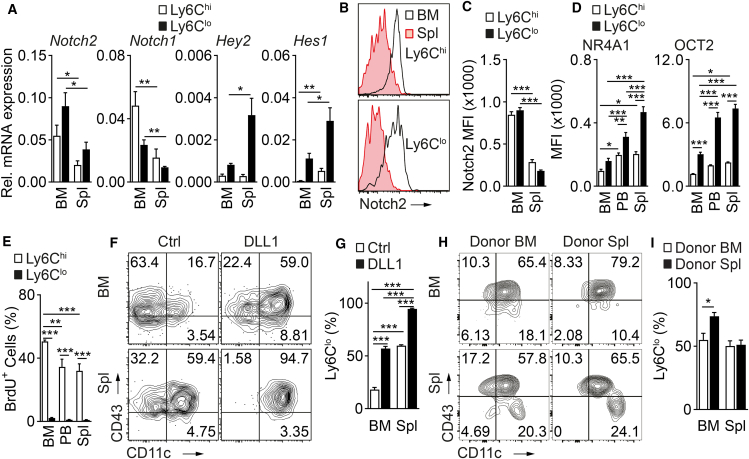
Monocyte subsets show compartment-specific differences in Notch signaling activity (A) Bar graphs showing relative expression of Notch receptors and Notch-regulated genes in Ly6C^hi^ and Ly6C^lo^ monocytes sorted from BM and Spl and analyzed by RT-qPCR. Data are pooled from three experiments; *n* = 3–5. (B and C) Representative flow cytometry histogram (B) and a bar graph (C) showing expression of the Notch2 receptor on the monocyte subsets. Data are representative of two independent experiments; *n* = 3. (D) Expression of monocyte signature transcription factors NR4A1 and OCT2 (encoded by *Pou2f2*) in monocyte subsets. Data are pooled from four experiments (*n* = 5/6). (E) Differential proliferation potential of monocyte subsets in organs as analyzed by BrdU incorporation. Bar graphs showing the relative frequency of BrdU^+^ cells. Data are pooled from three experiments (*n* = 6). (F and G) Representative flow cytometry plots (F) and bar graphs (G) depicting the relative frequency of Ly6C^lo^ monocytes in cultures. Data are representative of two independent experiments (*n* = 3). (H and I) Monocyte subsets show compartment-specific heterogeneity in conversion *in vivo*. Representative flow cytometry plots (H) and bar graphs depicting the relative frequency of donor-derived Ly6C^lo^ monocytes in donor total monocytes are shown. Data are pooled from two independent experiments (*n* = 6). (A, C, and I) Data are shown as mean ± standard error of the mean (SEM); ∗*p* < 0.05, ∗∗*p* < 0.01, ∗∗∗*p* < 0.001; two-tailed unpaired Student’s t test; (D, E, and G) Data are shown as mean ± SEM; ∗*p* < 0.05, ∗∗*p* < 0.01, ∗∗∗*p* < 0.001; one-way ANOVA with Bonferroni’s multiple comparison test.

To characterize monocyte subset heterogeneity in central (BM) and peripheral (spleen) compartments and the associated Notch signaling activity, we first sorted and analyzed prototypical Ly6C^hi^ and Ly6C^lo^ monocytes from BM and spleen. RNA and surface expression of receptors Notch2, involved in monocyte conversion,^13^ and Notch1 were reduced in the spleen compared to BM in both monocyte subsets (Figures 1A–1C), while Notch-regulated genes *Hey2* and *Hes1* were upregulated in spleen monocytes compared to BM (Figure 1A). Furthermore, levels of transcription factors NR4A1 and OCT2 in both monocyte subsets were higher in peripheral compartments (PB, spleen) compared to BM (Figure 1D), confirming a previous report.^4^

Higher Notch signaling activity was associated with lower proliferation behavior. After a single pulse of BrdU followed by overnight chasing, the frequency of BrdU^+^ Ly6C^hi^ monocytes was higher in BM than in spleen and PB, confirming previous studies (Figure 1E).^5^^,^^9^

We next tested *in vitro* whether the compartment source and the associated differences in endogenous Notch activity alter monocyte conversion response. We have shown previously that, *in vitro,* BM Ly6C^hi^ monocytes, in the presence of recombinant Notch ligand DLL1, convert to CD11b^+^Ly6C^lo/−^CD11c^+^CD43^+^ Ly6C^lo^ monocytes, consistent with patrolling monocytes.^13^^,^^27^ Compared to BM Ly6C^hi^ monocytes, spleen Ly6C^hi^ monocytes, which have higher endogenous Notch activity, showed higher conversion rates to Ly6C^lo^ monocytes without and with DLL1 stimulation (Figures 1F and 1G). Together, these data suggest compartment-specific differences in monocyte Notch activity and conversion behavior.

To assess the differentiation potential of Ly6C^hi^ monocytes derived from different compartments *in vivo*, we sorted Ly6C^hi^ monocytes from the BM and spleen of CD45.2^+^ CX_3_CR1-GFP^+^ donor mice, transferred them adoptively into congenic CD45.1^+^ mice and monitored the phenotype and fate of CD45.2^+^ Ly6C^hi^ progeny in recipients. Compared to donor monocytes from the BM, donor splenic monocytes converted at a higher rate to Ly6C^lo^ monocytes in recipient BM, which is in line with recent studies pointing to BM blood vessels being the place of conversion (Figures 1H and 1I).^33^ Thus, monocyte subsets show compartment-specific developmental heterogeneity linked with differences in intrinsic Notch signaling, specifically, monocytes from the periphery being more mature and ready for rapid differentiation.

### Extended monocyte subset characterization reveals differential role for Notch2 and Rbpj

**Figure 2:**
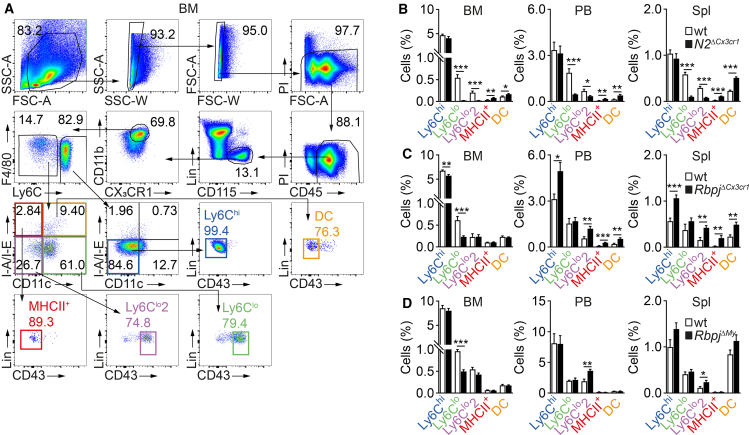
Discordant effects of deletion of Notch2 and Rbpj on monocyte subsets (A) Representative gating strategy for the definition and quantitative and qualitative analysis of monocyte subsets and DCs by flow cytometry in BM. Monocyte subsets were defined from the Lin^neg^CD45^+^CD115^+^CD11b^+^ gate after excluding doublets and dead propidium iodide^+^ (PI^+^) cells. (B–D) Relative frequency of different monocyte subpopulations and DCs in BM, PB, and Spl of *N2*^*ΔCx3cr1*^ (B), *Rbpj*^*ΔCx3cr1*^ (C), or *Rbpj*^*ΔMy*^ (D) mice and appropriate littermate controls (wt) are shown as mean ± SEM. (B–D) Data are pooled from two experiments, with *n* = 8 (B), *n* = 6–8 (C), and *n* = 6–8 (D) mice. (B–D) ∗*p* < 0.05, ∗∗*p* < 0.01, ∗∗∗*p* < 0.001; two-tailed unpaired Student’s t test. See also Figures S1 and S2.

To address the influence of Notch signaling on monocyte heterogeneity in compartments, we chose a strategy to analyze and compare conditional deletion of alleles of two principal Notch signaling components. These were targeted with myeloid Cre-driver strains that act either broadly or more restricted within the monocyte lineage. To this end, we analyzed mice with conditional deletion of *Notch2* or the Notch signaling transcriptional mediator *Rbpj* in monocytes and/or monocyte progenitors, by crossing mice bearing floxed *Notch2* or *Rbpj* with *Cx3cr1*^*Cre*9^ mice to obtain *N2*^*ΔCx3cr1*^ and *Rbpj*^*ΔCx3cr1*^ mice. Based on the broad expression profile of the *Cx3cr1* promoter, these lines target monocyte progenitors and their progeny, like mature monocytes and macrophages. As comparators, we also studied mice with *Lyz2*^*Cre*^-mediated targeting of *Notch2* and *Rbpj* (*N2*^*ΔMy*^ and *Rbpj*^*ΔMy*^, respectively^13^^,^^27^^,^^34^), which show Cre-activity from the mature Ly6C^hi^ monocyte stage onward.^9^^,^^13^ Using a conventional gating strategy, we defined different subsets of Lin^neg^CD11b^+^CD115^+^CX_3_CR1^+^ monocytes based on expression of Ly6C, CD43, CD11c, and I-A/I-E in different compartments (Figures 2A, S1A, and S1B).

We thus identified conventional (inflammatory) Ly6C^hi^ monocytes (Ly6C^hi^CD11c^neg^I-A/I-E^neg^CD43^neg^) and three other subsets of monocytes expressing low levels of Ly6C: two subsets of prototypical Ly6C^lo^ monocytes: (Ly6C^lo^: Ly6C^lo/−^CD11c^+^I-A/I-E^neg^CD43^+^) and (Ly6C^lo^2: Ly6C^lo/−^CD11c^neg^I-A/I-E^neg^CD43^+^) and MHCII^+^ CD43^neg^ monocytes (MHCII^+^: Ly6C^lo/−^CD11c^neg^I-A/I-E^+^CD43^neg^) and DC (Ly6C^lo/−^CD11c^+^I-A/I-E^+^CD43^neg^) (Figures 2A, S1A, and S1B; Table S1).

Based on this gating strategy, we performed a systematic quantitative analysis of BM, PB, and spleen monocyte subsets (Figures 2B–2D and S2A–S2L). *N2*^*ΔCx3cr1*^, compared to control mice, showed a marked reduction of Ly6C^lo^ and Ly6C^lo^2 monocytes in BM, PB, and spleen (Figures 2B, S2A–S2C, and S2J). The relative reduction was apparent in all compartments, being most prominent in BM (23.5- and 9.4-fold decrease of Ly6C^lo^ and Ly6C^lo^2 monocytes, respectively), while PB and spleen showed smaller reductions (Ly6C^lo^: 4.1-, and 5.9-fold; Ly6C^lo^2: 2.0-, and 3.7-fold). Furthermore, MHCII^+^ monocytes and DCs markedly increased in all three compartments (Figures 2B, S2A–S2C, and S2J). In contrast, *Rbpj*^*ΔCx3cr1*^ mice showed notably smaller changes in monocyte subsets, with only a slight reduction in Ly6C^lo^ monocytes (2.6-fold) in the BM (Figures 2C, S2D–S2F, and S2K). At the same time, the frequencies of Ly6C^lo^2, MHCII^+^ monocytes, and DC increased in PB and spleen but not in the BM. The frequency of Ly6C^hi^ monocytes was reduced in the BM but increased in the PB and spleen of *Rbpj*^*ΔCx3cr1*^ mice.

The changes observed in *Rbpj*^*ΔMy*^ mice, with myeloid targeting at the level of Ly6C^hi^ monocytes onward, were comparable to changes in *Rbpj*^*ΔCx3cr1*^ mice involving progenitor populations. There was a modest reduction of Ly6C^lo^ monocytes in the BM (Figures 2D, S2G–S2I, and S2L), whereas Ly6C^lo^2 cells were increased in PB and spleen. In contrast, Ly6C^hi^ and MHCII^+^ monocytes, as well as DCs remained unchanged compared to littermate controls (Figures 2D, S2G–S2I, and S2L). These quantitative data of Ly6C^lo^, Ly6C^lo^2, and MHCII^+^ monocytes and DCs demonstrate compartment-specific and disparate effects of targeting *Notch2* or *Rbpj* on monocyte subset regulation. *Notch2* deletion seems to impair Ly6C^lo^ and Ly6C^lo^2 monocyte development in all compartments tested and expand MHCII^+^ monocytes and DCs. In contrast, *Rbpj* deletion impairs Ly6C^lo^ monocytes only in the BM and expands Ly6C^lo^2 and MHCII^+^ monocytes and DCs in the periphery. Together, this may indicate prominent noncanonical effects in monocyte subset regulation.

### Notch2 and Rbpj differentially regulate monocyte phenotype and differentiation

We next performed systemic comparative analysis of monocyte subpopulation phenotypes in *N2*^*ΔCx3cr1*^, *Rbpj*^*ΔCx3cr1*^, *Rbpj*^*ΔMy*^, and *N2*^*ΔMy*^ mice to identify genotype-specific similarities and discrepancies.

**Figure 3:**
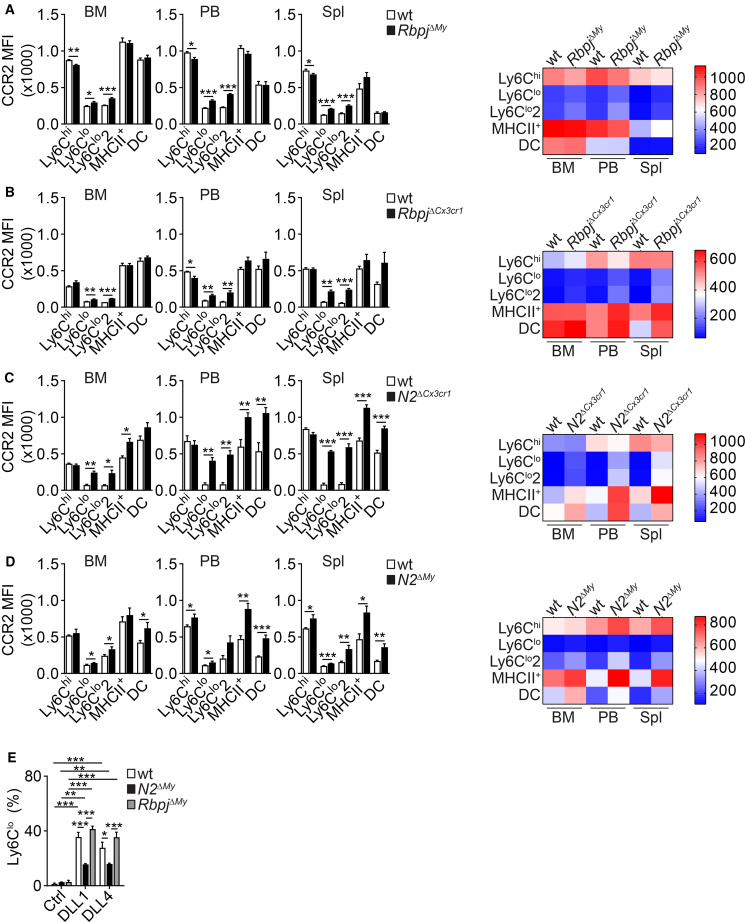
Comparative characterization of monocyte phenotype and proliferation (A–D) Expression of CCR2 on Ly6C^hi^, Ly6C^lo^, Ly6C^lo^2, and MHCII^+^ monocytes and DCs from *Rbpj*^*ΔMy*^ (A), *Rbpj*^*ΔCx3cr1*^ (B), *N2*^*ΔCx3cr1*^ (C), or *N2*^*ΔMy*^ (D) mice and (wt) controls defined by flow cytometry. Bar graphs and corresponding heat maps showing the mean fluorescence intensity (MFI) of CCR2 are depicted. Data are representative of two experiments [*n* = 3/5 (A), *n* = 3/3 (B), *n* = 3/5 (C), *n* = 5/3 (D)]. (E) Ly6C^hi^ monocytes show different conversion potential *in vitro*. *N2*^*ΔMy*^ - or *Rbpj*^*ΔMy*^ and wt control BM Ly6C^hi^ monocytes were cultured *in vitro* in the presence of Ctrl, DLL1, or DLL4 ligands, and the relative frequency of Ly6C^lo^ monocytes was determined using flow cytometry. Data are pooled from two experiments and shown as mean ± SEM (*n* = 6). (A–D) Data are shown as mean ± SEM; ∗*p* < 0.05, ∗∗*p* < 0.01, ∗∗∗*p* < 0.001; Student’s t test. (E) ∗*p* < 0.05, ∗∗*p* < 0.01, ∗∗∗*p* < 0.001; two-way ANOVA with Bonferroni’s multiple comparison test. See also Figures S3 and S4.

CCR2 is highly expressed in Ly6C^hi^ inflammatory monocytes, while Ly6C^lo^ patrolling monocytes are CCR2-negative in the steady state (Figures 3A–3D).^3^ Conditional deletion of *Rbpj* based on *Lyz2*^*Cre*^ targeting has been shown to upregulate CCR2 in Ly6C^lo^ monocytes, which has been suggested to influence cell distribution.^13^^,^^19^ Our extended comparative analysis of *Rbpj*^*ΔMy*^ mice confirmed moderate CCR2 upregulation in this subset in BM, PB, and spleen, which also affected Ly6C^lo^2 monocytes in these compartments (Figure 3A). *Rbpj*^*ΔCx3cr1*^ mice generally recapitulated this phenotype (Figure 3B). In contrast, *N2*^*ΔCx3cr1*^ mice showed marked CCR2 upregulation in all Ly6C^lo^ monocyte subsets, including MHCII^+^ monocytes and DCs, across all compartments, which was recapitulated in *N2*^*ΔMy*^ mice, but to a lesser extent (Figures 3C and 3D). Thus, deletion of *Notch2* shows stronger effects on CCR2 expression and involves more subpopulations than deletion of *Rbpj*, independent of targeting strategy.

Expression of the prototypical Ly6C^lo^ monocyte marker CD43^8^ was strongly reduced in Ly6C^lo^ monocytes from *N2*^*ΔCx3cr1*^ compared to *Rbpj*^*ΔCx3cr1*^ mice, while targeting of mature monocytes in *N2*^*ΔMy*^ and *Rbpj*^*ΔMy*^ mice did not affect CD43 expression (Figures S3A–S3D). The expression of NR4A1 was comparable between Notch component-mutant monocytes, as was the short-term BrdU incorporation used to measure proliferation *in vivo* (Figures S4A–S4D).

To test the response of mutant monocytes to specific Notch ligand stimulation and macrophage colony-stimulating factor (M-CSF), we performed *in vitro* culture experiments with sorted BM Ly6C^hi^ monocytes. Monocyte conversion *in vitro* was strongly affected by Notch receptor expression. The frequency of spontaneous conversion of Ly6C^hi^ monocytes into Ly6C^lo^ monocytes in the control culture was low in all genotypes (Figure 3E). DLL1- or DLL4-induced conversion was significantly reduced in Notch2-mutant monocytes while remaining unchanged in Rbpj-mutant monocytes (Figure 3E). Thus, DLL1 and DLL4 signaling drive monocyte conversion in a Notch2-dependent but in an Rbpj-independent manner, suggesting noncanonical effects.

### Notch2 and Rbpj regulate myeloid response in systemic inflammation

We next compared the response of myeloid cell populations to the inflammatory challenge in a TLR7-dependent model of inflammation in control and mutant strains. The TLR7 ligand Imiquimod (IMQ) induces expansion of Ly6C^lo^ patrolling monocytes in wt control mice in a Notch2-dependent manner, while *N2*^*ΔMy*^ mice respond with expansion of F4/80^hi^ MFs in PB, spleen, and aorta.^27^

**Figure 4:**
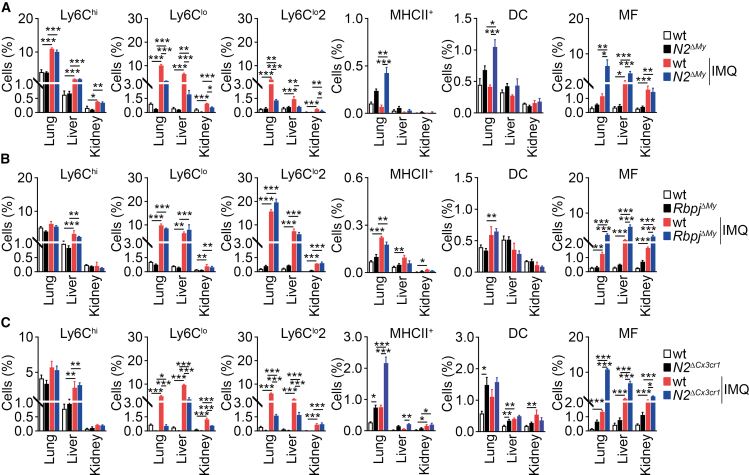
Notch2- and Rbpj-deficient mice show similarities and discrepancies in inflammatory myeloid response (A) Frequency of Ly6C^hi^, Ly6C^lo^, Ly6C^lo^2, and MHCII^+^ monocytes, DCs, or MFs in *N2*^*ΔMy*^ or control (wt) mice treated with IMQ or left without treatment. Bar graphs showing the relative frequency of cells in the lung, liver, and kidney. Data are pooled from two experiments (*n* = 3–7). (B) Frequency of Ly6C^hi^, Ly6C^lo^, Ly6C^lo^2, and MHCII^+^ monocytes, DCs, or MFs in *Rbpj*^*ΔMy*^ or control (wt) mice treated with IMQ or left without treatment. Bar graphs showing the relative frequency of cells in the lung, liver, and kidney. Data are pooled from two experiments (*n* = 3–6). (C) Frequency of Ly6C^hi^, Ly6C^lo^, Ly6C^lo^2, and MHCII^+^ monocytes, DCs, or MFs in *N2*^*ΔCx3cr1*^ or control (wt) mice treated with IMQ or left without treatment. Bar graphs showing the relative frequency of cells in the lung, liver, and kidney. Data are pooled from two experiments (*n* = 3–7). (A–C) Data are shown as mean ± SEM; ∗*p* < 0.05, ∗∗*p* < 0.01, ∗∗∗*p* < 0.001; two-way ANOVA with Bonferroni’s multiple comparison test.

We analyzed the frequency of extended monocyte subsets and macrophages in IMQ-treated *N2*^*ΔMy*^, *Rbpj*^*ΔMy*^, and *N2*^*ΔCx3cr1*^ mice, focusing on the lung, liver, and kidney (Figures 4A–4C). Comparing wt to *Notch2*-mutant mice, there was an expansion of Ly6C^lo^ and Ly6C^lo^2 monocytes in all organs of wt mice after IMQ treatment (Figures 4A and 4C), which was blunted in Notch2-deficient mice, which at the same time showed expansion of MHCII^+^ monocytes in the lungs (Figures 4A and 4C). The frequency of MF increased in IMQ-treated wt, *N2*^*ΔCx3cr1*^, or *N2*^*ΔMy*^ mice, but knock-out animals showed much higher numbers, especially in the lung and liver (Figures 4A and 4C). The frequency of Ly6C^hi^ monocytes did not differ between the wt and *Notch2* knockout mice but remained high after IMQ treatment in all animals (Figures 4A and 4C).

In *Rbpj*^*ΔMy*^ mice, Ly6C^lo^ and Ly6C^lo^2 monocytes expanded in the lung, liver, and kidney after IMQ treatment, but in contrast to Notch2-deficient mice, there was no difference between wt and knockout animals (Figure 4B). Similarly, MHCII^+^ monocytes did not expand in *Rbpj*^*ΔMy*^ mice treated with IMQ (Figure 4B). Changes in MF frequencies were comparable with Notch2-deficient strains, showing higher numbers in *Rbpj*^*ΔMy*^ animals compared to controls after IMQ treatment (Figure 4B). Thus, Notch2 and Rbpj control MF development in inflammatory conditions, while the development of Ly6C^lo^, Ly6C^lo^2, and MHCII^+^ monocytes is regulated via Notch2.

### Notch2 and Rbpj control late stages of monocyte lineage

We next aimed to characterize myeloid progenitor populations in Notch-deficient mice in relation to the targeting strategy. Using flow cytometry with an adapted conventional gating strategy^10^^,^^11^^,^^20^^,^^35^ in combination with unsupervised t-distributed stochastic neighbor embedding analysis (t-SNE), we identified early and late myeloid progenitors within Lin^neg^CD45^+^CD11b^lo/neg^ BM cells (Figures S5A–S5C; Table S2).

The monocyte lineage marker CX_3_CR1 showed preferential expression in MDP2 (Ly6C^+^ MDP), CDP, pre-monocytes (pMo1, pMo2), and mature monocytes compared to other progenitor subsets, suggesting that a *Cx3cr1*^*Cre*^-based targeting strategy may target the MDP2-dependent monocyte lineage as well as monocytes (Figure S5D).

Consistent with these findings of preferential lineage targeting, *N2*^*ΔCx3cr1*^ mice showed altered cell distribution in the mapped MDP2, pMo1, and pMo2 regions by t-SNE analysis (Figure S5C). Intriguingly, a subset of Ly6C^hi^ monocytes exhibited a partial reduction in Notch2 expression, yet the total cell counts remained unchanged (Figures S5E and 2B). Moreover, a pronounced decline in the prevalence of Notch2^+^ cells and Notch2 expression was observed across several populations, most striking in MDP2, but also in CDP, pMo1, pMo2, and mature monocytes. In contrast, the populations of CMP, GMP, MDP, and cMoP displayed minimal to no alterations in Notch2 surface expression (Figure S5E). Since Notch2 reduction in *N2*^*ΔCx3cr1*^ mice was highest in MDP2, this pattern suggests a hierarchical (preferential) targeting mechanism that favors MDP2 and its downstream progenitors over other progenitor populations such as GMP and cMoP (Figure S5E). In *N2*^*ΔMy*^ mice, *Notch2* deletion was milder and restricted to pMo1, pMo2, and mature monocytes (Figure S5F). Furthermore, despite subset-specific differences in *Notch2* targeting between *Cx3cr1*^*Cre*^- and *Lyz2*^*Cre*^-based strategies (Figures S5E and S5F), mutant mice had no quantitative alterations in early or late myeloid progenitors (Figures S5G, S5H, and S5K) and mature Ly6C^hi^ monocytes (Figure 2B),^27^ except a slight increase in the frequency of pMo2 in *N2*^*ΔMy*^ mice (Figure S5H). With the comparable reduction of Notch2 expression in pMo2 and Ly6C^hi^ monocytes in both models (Figures S5E and S5F), the reduction of Ly6C^lo^ monocyte numbers in *N2*^*ΔCx3cr1*^ mice was more severe (up to 23.5-fold) as compared to a 2-fold reduction in *N2*^*ΔMy*^ animals (Figure 2B).^27^ Similarly, there were no quantitative changes in Notch2 expression or early or late myeloid progenitors in *Rbpj*^*ΔCx3cr1*^ bone marrow cells except for a slight reduction of MDP2 (Figures S5I, S5J, and S5L). Consistent with these data, we confirmed efficient recombination of floxed *Notch2* and *Rbpj* loci using *Lyz2*^*Cre*^-based targeting in Ly6C^hi^ monocytes sorted from the BM and spleen of respective wt or knockout mice (Figure S6A).

Thus, from this comparative analysis of *Cx3cr1*^*Cre*^- and *Lyz2*^*Cre*^-based targeting approaches, we concluded that Notch2 or Rbpj control the later stages of myeloid development by regulating the fate of Ly6C^hi^ monocytes, with little to no effect on monocyte progenitor numbers. This regulation extends to their differentiation into mature phagocytes, including Ly6C^lo^, Ly6C^lo^2, and MHCII^+^ monocytes, MFs, and potentially to MoDCs.

### Single-cell RNA sequencing of monocyte subsets from BM and spleen

To extend our phenotypic observations, we labeled BM and spleen cells from control (wt), *Notch2*^*ΔCx3cr1*^, and *Rbpj*^*ΔCx3cr1*^ mice with different Hashtag oligos (HTOs) and antibodies, sorted myeloid cells (Figures S7A and S7B; Table S3), and performed comparative single-cell RNA sequencing (scRNA-seq) analysis.

**Figure 5:**
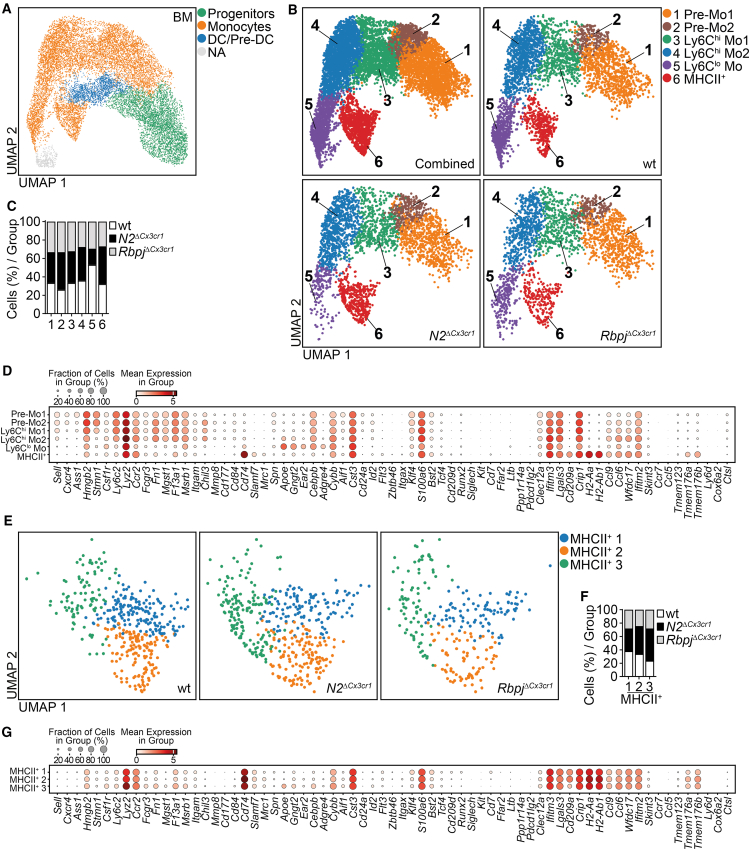
scRNA-seq analysis of Notch2- and Rbpj-deficient BM cells (A) UMAP plot of scRNA-seq data of sorted Lin^neg^Ly6A/E^neg^CD115^+^ BM cells with mapped populations concatenated from control (wt), *N2*^*ΔCx3cr1*^, and *Rbpj*^*ΔCx3cr1*^ mice (*n* = 14,774 cells). (B) BM monocyte clusters (from A) shown as concatenated or demultiplexed UMAP plots. (C) Ratio of cells per cluster normalized to input cell numbers corresponding to control, *N2*^*ΔCx3cr1*^, and *Rbpj*^*ΔCx3cr1*^ monocytes. (D) Bubble plot showing the expression of specific genes in the clusters from (B). The size of circles represents the percentage of cells and the intensity of color expression of specific gene within the cluster. (E) Subclustering of cluster #6 depicting heterogeneity within this cluster. Demultiplexed UMAPs show expansion and altered distribution of *N2*^*ΔCx3cr1*^ and *Rbpj*^*ΔCx3cr1*^ monocytes as compared to control cells in subclusters MHCII^+^ 1–3. (F) The ratio of cells per subcluster normalized to input cell numbers and corresponding to control, *N2*^*ΔCx3cr1*^, and *Rbpj*^*ΔCx3cr1*^ MHCII^+^ monocytes. (G) Bubble plot showing the expression of specific genes in the respective subclusters. The size of circles represents the percentage of cells and the intensity of color expression of specific gene within the cluster. See also Figures S7–S9, S12, and S14.

In the BM, on a two-dimensional Uniform Manifold Approximation and Projection (UMAP) plot, we discriminated progenitor populations, DC/Pre-DC, monocytes, and a small pool of unmapped cells (Figure 5A), which was confirmed by expression patterns of signature genes (Figure S8). Focusing on monocytes, a clustering analysis identified six clusters of cells with a set of hallmark genes (Figures 5B–5D). Clusters #3 and #4 (Ly6C^hi^ Mo1 and Ly6C^hi^ Mo2) expressed Ly6C^hi^ monocyte signature genes *Ly6c2*, *Lyz2*, *Ccr2*, *Fcgr3*, *Fn1*, *Mgst1*, *F13a1*, *Msrb1*, and *Chil3* (Figures 5B and 5D); clusters #1 and #2 (Pre-Mo1 and Pre-Mo2) expressed *Sell*, *Cxcr4*, *Ass1*, *Hmgb2*, and *Stmn1*, typical of pre-monocytes (Figures 5B and 5D); and cluster #5 expressed *Apoe*, *Gngt2*, *Ear2*, *Cebpb*, *Adgre4*, and *Cybb* corresponding to Ly6C^lo^ monocytes. Cluster #6 represented MHCII^+^ monocytes expressing *Cd74*, *H2-Aa*, *H2-Ab1*, *Cd209a*, *Tmem176a*, and *Tmem176b.* Of note, this cluster lacks expression of conventional DC genes *Itgax* and *Zbtb46* (Figures 5B and 5D) but partially expresses markers shared by Ly6C^lo^ and Ly6C^hi^ monocytes (*Apoe*, *Gngt2*, *Cebpb*, *Cybb*, *Fn1*, *F13a1*, *Msrb1*, and *Ly6c2)* (Figure 5D). At the same time, *Cd74* expression was also observed in a few cells of clusters #5, Ly6C^lo^ monocytes, and #4, Ly6C^hi^ Mo2 (Figure 5D). When we compared the control and knockout cells, we found that the frequency of cluster #6 (MHCII^+^ monocytes) was slightly increased in *N2*^*ΔCx3cr1*^ mice, and cluster #5 (Ly6C^lo^ monocytes) was reduced in *N2*^*ΔCx3cr1*^ and *Rbpj*^*ΔCx3cr1*^ BM (Figures 5B and 5C), while other clusters showed no quantitative changes. Thus, we confirmed the results observed by flow cytometry analysis: a reduction of patrolling monocytes in Notch-signaling-deficient mice and expansion of MHCII^+^ monocytes in *N2*^*ΔCx3cr1*^ mouse BM.

We next focused on comparative analysis of MHCII^+^ monocytes in cluster #6, which on the UMAP plot was subdivided into three subgroups MHCII^+^ 1–3 (Figures 5E–5G). Clustering analysis revealed that cluster MHCII^+^ 3 was larger in *N2*^*ΔCx3cr1*^ mice (Figures 5E and 5F), expressing markers of Ly6C^lo^ and Ly6C^hi^ monocytes, such as *Apoe*, *Gngt2*, *Cebpb*, *Cybb*, *F13a1*, *Fn1*, *Msrb1*, *Ccr2*, and *Lyz2* (Figures 5G and S9), but lacking expression of *Spn* (encoding CD43). All three subgroups of MHCII^+^ monocytes expressed *Cd74*, *H2-Aa*, *H2-Ab1*, and *H2-Eb1*, markers consistent with DCs, but lacked *Zbtb46* and *Itgax*. Additionally, a minority of cells in subcluster MHCII^+^ 3 also expressed low levels of *Cd209a* and *Flt3*, which distinguishes this cluster from the other two subclusters (Figures 5G and S9). This demonstrates that MHCII^+^ monocytes exhibit heterogeneity within the BM and share markers of DCs and Ly6C^lo^ monocytes.

**Figure 6:**
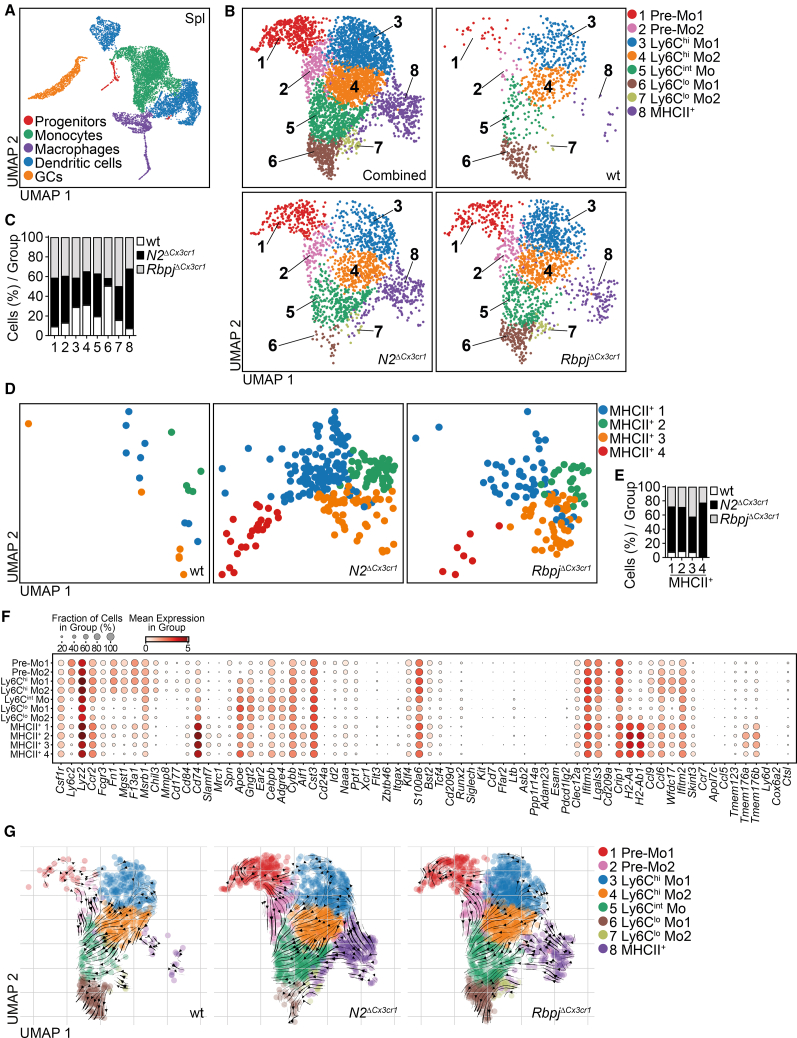
scRNA-seq analysis of Notch2- and Rbpj-deficient spleen myeloid cells (A) UMAP plot of scRNA-seq data of sorted live Lin^neg^ spleen myeloid cells with mapped populations concatenated from control, *N2*^*ΔCx3cr1*^, and *Rbpj*^*ΔCx3cr1*^ mice (*n* = 9,230 cells). (B) Spleen monocyte clusters [from (A)] shown as concatenated or demultiplexed UMAP plots. (C) The ratio of cells per cluster normalized to input cell numbers corresponding to control, *N2*^*ΔCx3cr1*^, and *Rbpj*^*ΔCx3cr1*^ monocytes. (D) Subclustering of cluster #5 depicting heterogeneity within this cluster. Demultiplexed UMAPs show expansion and altered distribution of *N2*^*ΔCx3cr1*^ and *Rbpj*^*ΔCx3cr1*^ monocytes as compared to control cells in subclusters MHCII^+^ 1–4. (E) The ratio of cells per subcluster normalized to input cell numbers. (F) Bubble plot showing the expression of specific genes in the respective monocyte clusters from (B) and subclusters MHCII^+^ 1–4 from (D). The size of circles represents the percentage of cells and the intensity of color expression of specific gene within the cluster. (G) Demultiplexed UMAP plots showing RNA velocity analysis of spleen monocytes. See also Figures S7, S10, S11, S13, and S14.

In the spleen, we identified progenitors, DCs, monocytes, MFs, and granulocytes (GCs) (Figure 6A), which was confirmed by signature gene expression (Figure S10). Clustering analysis of monocytes identified eight clusters of cells (Figures 6B and 6C): clusters #1 and #2—pre-monocytes (Pre-Mo1 and Pre-Mo2); clusters #3 and #4—Ly6C^hi^ monocytes (Ly6C^hi^ Mo1 and Ly6C^hi^ Mo2); cluster #5—Ly6C intermediate monocytes (Ly6C^int^ Mo); clusters #6 and #7—Ly6C^lo^ monocytes (Ly6C^lo^ Mo1 and Ly6C^lo^ Mo2); and cluster #8—MHCII^+^ monocytes.

Quantitative analysis of control and knockout cells showed a reduction of cluster #6 (Ly6C^lo^ Mo1) in *N2*^*ΔCx3cr1*^ and *Rbpj*^*ΔCx3cr1*^ mice (Figures 6B and 6C), comparable to findings in BM above. At the same time, Pre-Mo1, Pre-Mo2, Ly6C^int^ Mo, and MHCII^+^ monocytes were expanded in both the knockout strains (Figures 6B and 6C). Additional analysis and quantification of cluster #8 (MHCII^+^ monocytes) separated four subclusters: MHCII^+^ 1–4 (Figures 6D, 6E, and S11). Gene expression analysis identified expression patterns similar to BM within the monocyte clusters and MHCII^+^ subclusters. Ly6C^hi^ Mo1 and Ly6C^hi^ Mo2 expressed hallmark genes (*Ly6c2*, *Lyz2*, *Ccr2*, *Fcgr3*, *Fn1*, *Mgst1*, *F13a1*, *Msrb1*, *Chil3*) of Ly6C^hi^ monocytes while Pre-Mo1 and Pre-Mo2 expressed *Hmgb2*, *Stmn1*, *S100a4*, and *S100a6* additionally (Figures 6F and S10). Ly6C^int^ Mo, Ly6C^lo^ Mo1, and Ly6C^lo^ Mo2 expressed *Apoe*, *Gngt2*, *Ear2*, *Cebpb*, *Adgre4*, and *Cybb*; however, Ly6C^int^ Mo also maintained higher *Ly6c2*, *Ccr2*, *Fn1*, and *Mgst1* expression (Figures 6F and S10). Subclusters MHCII^+^ 1–4 expressed high levels of *Cd74*, *H2-Aa*, *H2-Ab1*, *Tmem176a*, and *Tmem176b* but mainly lacked *Itgax*, *Zbtb46*, *Flt3*, and *Cd209a* or other markers of conventional DCs (Figures 6F and S11). At the same time, the mean expression of these markers varied within MHCII^+^ subclusters 1–4. Interestingly, *Apoe*, *Gngt2*, *Cebpb*, *Adgre4*, and *Cybb*, typical markers of Ly6C^lo^ monocytes, and *Fn1*, *F13a1*, *Msrb1*, *Lyz2*, and *Ly6c2*, markers of Ly6C^hi^ monocytes, were also expressed to varying degrees in MHCII^+^ 1–4 (Figures 6F and S11), showing distinctions between subclusters. Notably, low-level expression of *Cd74* and, to a lesser extent, *H2-Aa* and *H2-Ab1*, was seen in Ly6C^hi^-, Ly6C^int^-, and Ly6C^lo^ Mo clusters (Figure 6F).

Comparative analysis of Ly6C^hi^ Mo2, Ly6C^lo^ Mo, and MHCII^+^ subsets in BM and Ly6C^hi^ Mo1, Ly6C^hi^ Mo2, Ly6C^int^ Mo, Ly6C^lo^ Mo1, and MHCII^+^ subsets in spleen revealed Notch2- and Rbpj-dependent changes in expression of transcription factors linked with myeloid differentiation (Figures S12A and S13A) as well as Notch-signaling-related genes in respective knockout animals (Figures S14A and S14B).

Furthermore, RNA velocity analysis showed at least two potential developmental trajectories of the cells: one common pathway from Ly6C^hi^ Mo1/Ly6C^hi^ Mo2 toward Ly6C^int^ Mo to Ly6C^lo^ Mo1, present regardless of Notch-status, and a second pathway from Ly6C^hi^ Mo1 toward MHCII^+^ monocytes, which was most prominent in *N2*^*ΔCx3cr1*^ mice (Figure 6G).

Thus, scRNA-seq analysis of BM and spleen myeloid cells indicates considerable heterogeneity of monocytes and corroborates differences between Notch2- and Rbpj-deficient mice.

### Notch controls heterogeneity and plasticity of Ly6C^hi^ monocyte subtypes

We next investigated the plasticity of Ly6C^hi^ inflammatory monocyte subsets and the potential impact of Notch signaling. To this end, we analyzed the developmental potential of GC-like monocytes (potentially derived from GMP) and DC-like monocytes (potentially derived from MDP)^20^^,^^21^ in a defined *in vitro* culture system with sorted Ly6C^hi^ monocyte subsets (Figures S15A and S15B). We purified CD177^+^Lin^neg^CD11b^+^CD115^+^Ly6C^hi^ GC-like monocytes and CD319^+^Lin^neg^CD11b^+^CD115^+^Ly6C^hi^ DC-like monocytes^22^ from wt or *N2*^*ΔCx3cr1*^ BM as cell input (Figure S15A). These cells were cultured *in vitro* in the presence of M-CSF, with or without recombinant Notch ligands DLL1 or DLL4, and differentiation into Ly6C^lo^ monocytes (CD11b^+^Ly6C^lo/−^CD11c^+^CD43^+^), MoDCs (CD11b^+^Ly6C^lo/−^CD11c^+^I-A/I-E^+^), or MHCII^+^ monocytes (CD11b^+^Ly6C^lo/−^CD11c^neg^I-A/I-E^+^) was quantified (Figure S15C).

**Figure 7:**
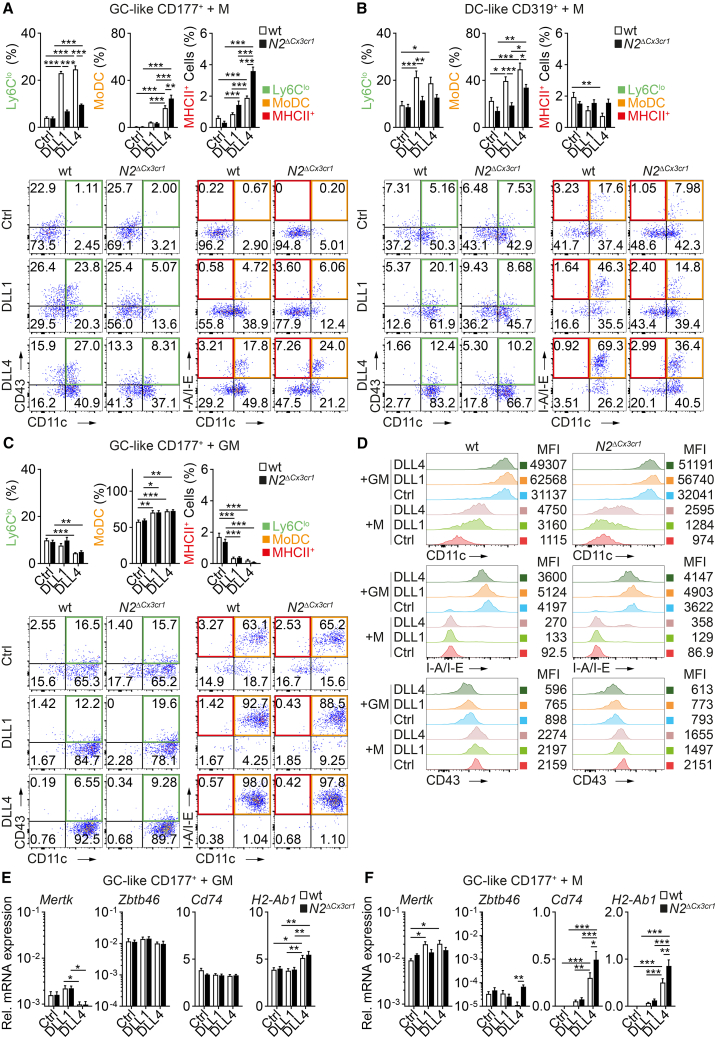
Notch2 controls DC-like and GC-like monocyte differentiation *in vitro* (A and B) GC-like and DC-like Ly6C^hi^ monocytes show differences in Notch-dependent conversion *in vitro*. GC-like CD177^+^ (A) or DC-like CD319^+^ (B) Ly6C^hi^ monocytes were sorted from wt or *N2*^*ΔCx3cr1*^ BM and cultured *in vitro* in the presence of Ctrl, DLL1, or DLL4 ligands and M-CSF (+M). The relative frequency of Ly6C^lo^ monocytes, MHCII^+^ monocytes, and MoDCs in the cultures (depicted as mean ± SEM) and representative flow cytometry plots are shown (see also Table S4). (A and B) Data are pooled from four experiments (*n* = 8–12). (C) GC-like Ly6C^hi^ monocytes differentiate into MoDC *in vitro* under the influence of GM-CSF (+GM). GC-like CD177^+^ Ly6C^hi^ monocytes sorted from wt or *N2*^*ΔCx3cr1*^ BM were cultured *in vitro* in the presence of Ctrl, DLL1, or DLL4 ligands and GM-CSF. The relative frequency of Ly6C^lo^ and MHCII^+^ monocytes and MoDCs (depicted as mean ± SEM) and representative flow cytometry plots are shown. Data are pooled from three experiments (*n* = 9). (D) Flow cytometry histogram plots (and MFI) showing expression of respective surface markers in *in vitro* converted cells. (E and F) Expression of monocyte/macrophage- and DC-marker genes in converted cells. wt or *N2*^*ΔCx3cr1*^ GC-like Ly6C^hi^ monocytes were cultured on immobilized Notch ligands and in the presence of GM-CSF (E) or M-CSF (F), respectively. Converted cells were harvested, and gene expression was analyzed using RT-qPCR. Data are pooled from two experiments (*n* = 8). (A–C, E, and F) Data are shown as mean ± SEM; ∗*p* < 0.05, ∗∗*p* < 0.01, ∗∗∗*p* < 0.001; two-way ANOVA with Bonferroni’s multiple comparison test. See also Figure S12 and Table S4.

GC-like Ly6C^hi^ monocytes from wt mice, when cultured under control conditions without Notch ligand stimulation, had a low endogenous differentiation potential into any target cell type (ca 6%) (Figure 7A; Table S4). DLL1 promoted preferential differentiation into Ly6C^lo^ monocytes (23%), with only a small fraction of cells appearing as MoDC or MHCII^+^ monocytes, and this was dependent on functional Notch2. Similarly, DLL4 also promoted preferential differentiation into Ly6C^lo^ monocytes (25%) in a Notch2-dependent manner, but a 4-fold higher number of cells differentiated into MoDC (17%), which was further increased in the absence of functional Notch2 (25%, Figure 7A; Table S4). In contrast, DC-like Ly6C^hi^ monocytes from wt mice, when cultured under control conditions without Notch ligand stimulation, had a higher endogenous differentiation potential into target populations (34%), preferentially into MoDC (23%) and, to a lesser extent, Ly6C^lo^ monocytes (9%). DLL1 promoted the preferential differentiation into MoDC (40%) and Ly6C^lo^ monocytes (21%), which was dependent on Notch2 (Figure 7B; Table S4). Furthermore, DLL4 also enhanced preferential differentiation into MoDC (49%) and Ly6C^lo^ monocytes (19%), but this was only partially dependent on Notch2 (Figure 7B; Table S4).

Differentiation into MHCII^+^ monocytes was overall a rare event but was most prominent in GC-like monocytes from *N2*^*ΔCx3cr1*^ mice cultured on DLL4 (8-fold increase over baseline; Figure 7A). A culture system using CD74-based sorting into GC- (CD74^neg^) and DC-like (CD74^+^) Ly6C^hi^ monocytes (Figure S15B) resulted in essentially comparable findings (Figures S15D and S15E).

Together, these observations demonstrate a degree of plasticity within both subsets of Ly6C^hi^ monocytes but preferential differentiation of GC-like monocytes into Ly6C^lo^ monocytes with both ligands, while DC-like monocytes tend toward MoDC development, particularly with DLL stimulation and in the absence of Notch2. These data also suggest that MHCII^+^ monocytes are predominantly derived from GC-like monocytes and are highly dependent on the Notch ligand DLL4.

To evaluate the influence of growth-factor context, we cultured GC-like Ly6C^hi^ monocytes in the presence of GM-CSF (GM) and analyzed the phenotype of converted cells. Regardless of genotype or Notch ligand stimulation, the majority of cells differentiated into MoDC, while only a small fraction converted into Ly6C^lo^ monocytes or MHCII^+^ monocytes (Figures 7C and S15F). Furthermore, compared to M-CSF cultured cells, expression of CD11c and I-A/I-E was higher, while CD43 expression was lower (Figure 7D). In gene expression analysis, GM-CSF induced strong expression of *Zbtb46*, a hallmark transcription factor of DCs, *Cd74*, and *H2-Ab1* in a Notch-independent manner, but expression of *Mertk* was low (Figure 7E). Interestingly, *Mertk* downregulation by GM-CSF cultures was most prominent in the presence of DLL4, which coincided with significantly higher expression of *H2-Ab1* as compared to DLL1 or Ctrl conditions, suggesting that DLL4-dependent signaling in combination with GM-CSF drives monocyte differentiation toward MoDCs (Figure 7E). In contrast, M-CSF induced high levels of *Mertk* but low levels of *Zbtb46*, *Cd74*, and *H2-Ab1*, indicating an active monocyte-macrophage differentiation program (Figure 7F). At the same time, *Mertk* expression in M-CSF cultures was higher in the presence of DLL1 and DLL4 but partially reduced in Notch2-deficient cells (Figure 7F). Furthermore, cells cultured in the presence of DLL4 also expressed high *Cd74* and *H2-Ab1*, with highest levels observed in cells from *N2*^*ΔCx3cr1*^ mice (Figure 7F). This suggests that GM-CSF and M-CSF promote distinct programs of cell differentiation, leading to MoDC or monocyte/macrophage development, respectively.

To summarize, both GC-like and DC-like monocytes possess plasticity and can lead to alternative cell fates but to different extents. Specifically, during their development into monocytes/macrophages or MoDCs, the fate of GC-like Ly6C^hi^ monocytes might be co-regulated by specific DLL1-Notch2 or DLL4-Notch2 signaling pairs in combination with specific growth factors such as M-CSF or GM-CSF.

Thus, in general, Notch2 signaling controls monocyte conversion, monocyte surface phenotype, and function in a canonical, Rbpj-dependent and noncanonical, Rbpj-independent manner, thereby contributing to organ-specific heterogeneity and developmental plasticity of monocyte pools (Figure S16A). Such control depends on specific Notch ligands and respective growth factors.

## Discussion

Using an extended monocyte subset analysis, we show that monocyte heterogeneity in distinct compartments may be more complex than the so far perceived subsets of Ly6C^hi^ and Ly6C^lo^ monocytes or GC-like and DC-like monocytes. The heterogeneity includes differences in cell phenotype and behavior, which at least in part is linked to Notch signaling and affects central (bone marrow) and peripheral (splenic) Ly6C^hi^ monocytes as well as several subsets with low Ly6C^lo^ expression. Ly6C^hi^ monocytes residing in the spleen have lower proliferation potential than their BM counterparts, show higher endogenous Notch signaling activity, and consequently, are more prone to Notch-dependent monocyte conversion. This heterogeneity is in line with previous reports describing differences in monocytes between BM and periphery at the transcriptomic and promotor activity level, despite gross phenotypic similarities of cells between compartments.^4^ Namely, the Ly6C^lo^ monocyte pool consists of prototypical Ly6C^lo^ monocytes, Ly6C^lo^2, and MHCII^+^ monocytes. From extended monocyte characterization and systematic comparative analysis of different Cre-based Notch2- and Rbpj-mutant strains, prototypical Ly6C^lo^ and Ly6C^lo^2 subsets seem to be closely related cells, probably representing different stages along terminal maturation. This is suggested by gross similarities in the surface phenotype, developmental Notch-signaling dependency, and spatial distribution patterns, which also suggest strong functional overlap, e.g., typical vessel-patrolling monocytes. The MHCII^+^ monocyte subset, in contrast, is distinct from prototypical Ly6C^lo^ monocytes. It features markers of Ly6C^lo^ monocytes and DCs and shows a distinct, and sometimes opposite response to Notch2 deficiency. However, function and cell fate are currently unclear and require further studies.

The Notch2 receptor influences the heterogeneity of monocytes in an Rbpj-dependent (canonical) or -independent (noncanonical) manner, demonstrated by similarities and differences in mutant-strain comparisons (Figure S16A). They define the developmental trajectories of Ly6C^hi^ monocytes by controlling monocyte differentiation into distinct phenotypic and functional subsets in different compartments. Specifically, Rbpj regulates the expression of CCR2 on Ly6C^lo^ and Ly6C^lo^2 monocytes, which is linked to monocyte distribution. These data align with a recent study showing CCR2-dependent rapid egress of Ly6C^lo^ monocytes from BM to the periphery in *Rbpj*-deficient mice.^19^ CCR2 is also upregulated in *Notch2*-deficient Ly6C^lo^ and Ly6C^lo^2 monocytes, suggesting that CCR2 expression is under tight control of canonical Notch2 signaling in these cells. However, in *Notch2*-deficient MHCII^+^ monocytes and DCs, CCR2 expression is regulated in an Rbpj-independent manner, further supporting separation of these cells into distinct subsets. The influence of Notch2 on monocytes is more complex and extends beyond CCR2 regulation, as it regulates monocyte conversion and monocyte cell fate (Figure S16A). *Notch2* deficiency causes quantitative and qualitative changes in monocyte subsets in a non-canonical, Rbpj-independent manner. Specifically, Notch2 controls the development of Ly6C^lo^, Ly6C^lo^2, and MHCII^+^ monocytes *in vivo* or Ly6C^lo^, MHCII^+^ monocytes, and MoDCs *in vitro* in a Notch-ligand-dependent manner.

In inflammatory conditions, Notch signaling regulates monocyte cell fate on several levels, which shapes tissue phagocyte pools. Loss of *Notch2* or *Rbpj* increases the frequency of F4/80^hi^ MFs in the lung, liver, and kidney. However, MHCII^+^ monocytes expand mainly in *Notch2*-deficient, but not in *Rbpj*-deficient, mice. These data are in line with a recent study showing an increased number of BM-monocyte-derived interstitial lung MFs after Notch2 blockade during bleomycin-induced fibrosis.^36^ It is important to mention that Notch2- or Rbpj-mediated expansion of MFs in inflammatory conditions is not an isolated phenomenon and is observed at least in the liver, lung, and kidney. In agreement with this, Rbpj was shown to be implicated in monocyte-to-macrophage transition and the development of protective Ly6C^lo^ monocytes in liver diseases.^29^ Notch-signaling-axis-dependent regulation of Ly6C^hi^ inflammatory monocyte cell fate and development of Ly6C^lo^, MHCII^+^ monocytes, and MFs in inflammatory conditions might be regulated through the Dll1/Dll4 ligands expressed in specific tissue niches. This may define the intensity and the character of the response, which needs further studies.

It is still unclear how ontogeny and plasticity contribute to heterogeneity of Ly6C^hi^ monocytes. On one hand, Ly6C^hi^ monocyte subsets could be irreversibly committed to develop into distinct terminally differentiated phagocytes, such as MFs, Ly6C^lo^, Ly6C^lo^2, and MHCII^+^ monocytes or MoDCs independent of niche signals. Alternatively, monocyte subsets might possess inherent plasticity to shape their differentiation path depending on environmental conditions and signals. In this context, our scRNA-seq data show a Notch2- and partially Rbpj-dependent switch of monocyte developmental trajectories from Ly6C^hi^ monocytes toward Ly6C^lo^ or MHCII^+^ monocytes. *In vitro* studies using recombinant ligands DLL1 and DLL4 in combination with specific growth factors further confirmed these data. Specifically, Notch2 regulated GC- or DC-like monocyte differentiation toward Ly6C^lo^, MHCII^+^ monocytes, or MoDCs *in vitro* in a Notch-ligand-dependent manner under the influence of M-CSF. The presence of two characterized Ly6C^hi^ monocyte subsets (GC- and DC-like) may promote rapid responses to various stressors. However, GC-like Ly6C^hi^ monocytes possess intrinsic plasticity, and while their *in vitro* endogenous differentiation potential is low, they readily differentiate into phenotypically different cells in response to specific signaling cues. Specifically, the influence of M-CSF in combination with either Delta-ligand promotes prototypical Ly6C^lo^ monocyte differentiation, which is mediated by Notch2. The combination of M-CSF with DLL4 in the absence of Notch2 promotes the development of MHCII^+^ monocytes and MoDC. In contrast, the presence of GM-CSF promotes MoDC development independent of Notch2.

In our previous work, we found that monocyte conversion and Ly6C^lo^ monocyte development is driven by DLL1-Notch2 signaling.^13^ The current study extends previous observations on the ligand Dll4, showing that it can substitute for Dll1 and promote Ly6C^lo^ monocyte development equally in the presence of M-CSF *in vitro*. Dll4 mediates the conversion of DC-like and GC-like Ly6C^hi^ subsets, but only GC-like monocytes depended on Notch2. In addition, the effect of Dll4 is more heterogeneous as it drives the development of MHCII^+^ monocytes and MoDCs parallel to Ly6C^lo^ monocytes, while Dll1 promotes the conversion of GC-like Ly6C^hi^ monocytes predominantly into Ly6C^lo^ monocytes.

Liu et al. identified a new subset of conventional DCs—DC3,^7^ which develops from Ly6C^+^ proDC3 and shares some similarities with DC-like monocytes or cDC2. Similarly, a subset of cDC2 (cDC2a or CD24^+^ cDC2) is regulated in Notch2- and Rbpj-dependent manner,^32^^,^^37^ while the cDC2b (CD24^neg^) subset appears to be Notch-independent. However, MHCII^+^ monocytes, in contrast to cDC2a, cDC2b, or DC3, expand in Notch2-deficient mice and are phenotypically distinct to them, e.g., expression of CD11c. Thus, MHCII^+^ monocytes are potentially derived from GC-like Ly6C^hi^ monocytes, are separated from conventional DCs, and exist independently. In line with these data, we confirmed the development of MHCII^+^ monocytes from GC-like Ly6C^hi^ monocytes *in vitro* in a Notch2-dependent manner and in the presence of DLL4 and M-CSF.

Development of prototypical Ly6C^lo^ monocytes from Ly6C^hi^ monocytes requires *Nr4a1*, *Bcl6*, *Irf2*, and *Cebpb*^4^^,^^25^ and is DLL1-Notch2-dependent,^13^ while Notch2-independent Ly6C^lo^ monocytes can arise in the absence of *Cebpa,* potentially from myeloid progenitors.^26^ Recently, it was shown that induced MHCII^+^ Ly6C^lo^ monocytes expand by NOD2-agonist treatment and were involved in anti-tumor immunity.^38^ These cells, present in *Nr4a1*-deficient mice and proposed to be Notch2-independent, further confirmed the heterogeneity of Ly6C^lo^ monocytes. However, the phenotype of the induced population differs from the Notch2-dependent MHCII^+^ monocytes described in our study, the latter lacking CD11c and CD43, two markers of Ly6C^lo^ monocytes. Further studies are necessary to dissect the relationship between NOD2-agonist-dependent induced MHCII^+^ monocytes and MHCII^+^ monocytes expanded in Notch2-deficient conditions and described in the current manuscript.

Modulation of myeloid Notch signaling, thereby affecting monocyte fate, phenotype, and function, might be of high clinical importance, as monocytes are one of the first-line immune defense cells involved in acute or chronic inflammation. In line with these data, several mouse and human studies revealed the role of Notch2 or Rbpj in the regulation of monocytes in pathological conditions. Particularly, targeting of Rbpj in mice by specific inhibitors might ameliorate metabolic-dysfunction-associated steatohepatitis, potentially due to expansion of angioprotective Ly6C^lo^ patrolling monocytes.^29^ NOD2-induced nonclassical monocytes are believed to develop in the absence of Notch2 signaling and mediate protection against cancer metastases.^38^ Our observation that MHCII^+^ monocytes expand in Notch2-deficient mice in steady-state conditions might also be in line with NOD2-dependent inducible monocytes and potentially open new treatment options by modulating monocyte Notch2 signaling in cancer patients. Considering that these cells express MHCII, they might contribute to tumor antigen presentation. However, whether such events have tolerogenic or immunogenic consequences may depend on their functional status or the tumor microenvironment. Nevertheless, prior to clinical applications, additional studies defining the role of Notch2 and Rbpj in human monocyte conversion and regulation are needed; similarly, the human equivalent of MHCII^+^ monocytes yet needs to be identified. Crosstalk of endothelial cells with monocytes potentially by DLL4-NOTCH2 signaling is probably involved in antibody-mediated rejection after cardiac transplantation in patients, opening another avenue for clinical application of Notch targeting.^39^ Similarly, changes in expression of notch ligands, specifically Dll4, are observed in endothelial cells during sepsis and in the atherosclerotic plaques.^40^^,^^41^ They may regulate the cell fate of Ly6C^hi^ monocytes circulating in the blood and homing to the inflamed or altered vessel wall. Such mechanism can control the development of patrolling, MHCII^+^ monocytes, and macrophages and might determine the onset and pathogenesis of vascular diseases. Therefore, manipulating Notch signaling by targeting selective Notch ligands in the blood vessels or Notch receptors on inflammatory monocytes could be used to modulate monocyte differentiation programs and thus represent a potential treatment option. Recent study has shown that NOTCH2 expression in monocytes predicts interferon β (IFN-β) immunogenicity in patients with multiple sclerosis and hence might be involved in autoimmune or inflammatory diseases.^42^

Taken together, our study shows the heterogeneity and plasticity of monocyte subsets and reveals the differential contribution of Notch2 and Rbpj signaling.

### Limitations of the study

Our work has technical and model-specific limitations. We used mouse as a model organism to study the niche-specific heterogeneity and developmental plasticity of monocytes and regulation by Notch. These results, therefore, need further confirmation using human systems. Along this line, Ly6C^lo^, Ly6C^lo^2, and MHCII^+^ monocyte subsets, MFs, and DCs differ across both species. The human equivalents of these cells still need to be identified and confirmed precisely. Thus, the origin, developmental regulation, and the function of these cells are also still the subject of current studies, both in mice and in humans. Similarly, targeting of Notch2 and Rbpj using a Cre-mediated deletion strategy does not generate complete knockout of these genes in monocytes, monocyte precursors, and monocyte descendants, thereby complicating interpretation of the results, especially in inflammatory conditions. An alternative approach could be to use pharmacological inhibitors or antibodies targeting Notch signaling components. However, specificity of targeting still remains questionable. Furthermore, GC-like monocyte plasticity, particularly the generation of MoDCs, and the influence of Notch signaling and growth factors were only observed *in vitro*. Therefore, additional *in vivo* studies are needed to elucidate the developmental potential and molecular mechanisms. Likewise, our proof-of-principle *in vitro* studies were limited to Notch signaling and two growth factors, M-CSF and GM-CSF, but other signaling cascades or monocyte growth factors may also play important roles in regulating monocyte heterogeneity and plasticity.

## Resource availability

### Lead contact

Requests for resources and reagents, as well as for further information, should be directed to and will be fulfilled by the lead contact, Florian Limbourg (limbourg.florian@mh-hannover.de).

### Materials availability

Reagents generated in this study are available from the lead contact upon request after completion of the respective material transfer agreements.

### Data and code availability

• Data supporting the findings in this study are present within the article and supplemental information. Data from scRNA-seq are deposited to NCBI’s Gene Expression Omnibus under the accession number GEO: GSE289018. https://www.ncbi.nlm.nih.gov/geo/query/acc.cgi?acc=GSE289018.
• The original code is deposited under https://github.com/sgaedcke/Monocyte_heterogeneity_notch.
• All additional information necessary to reanalyze the data in this paper will be provided by the lead contact upon request.

## Acknowledgments

We thank the Central Animal Facility, Research Core Facility Cell Sorting, and Research Core Unit Genomics of 10.13039/501100005624MHH for excellent support. Funded by grants from DFG (GA 2443/3-1 and GA 2443/2-1) to J.G., DFG (Li948-10/1 and Li948-7/1) to F.P.L., and DFG (KA 5549/2-1) to T.K. Y.X. was supported by a grant from the 10.13039/501100004543China Scholarship Council.

## Author contributions

Y.X., T.K., S.S., J.G., and F.N.S. performed the experiments; Y.X., T.K., J.G., and F.P.L. designed experiments and analyzed data; S.G. and A.C.J. analyzed sequencing data; S.H., O.J.M., M.L., H.H., and K.S.-O. provided necessary resources, materials, or animals; Y.X., T.K., J.G., and F.P.L. wrote and edited the manuscript; all authors corrected the manuscript; J.G. and F.P.L. conceived and directed the study.

## Declaration of interests

The authors declare no competing interests.

## STAR★Methods

### Key resources table

**Table:** 

REAGENT or RESOURCE	SOURCE	IDENTIFIER
**Antibodies**		

Monoclonal Anti-Mouse BrdU Alexa Fluor® 647	BD Biosciences	Cat#560209; Clone 3D4; RRID: AB_1645615
Mouse Monoclonal Anti-mouse CD45.1 APC	BioLegend	Cat#110713; Clone A20; RRID: AB_313502
Mouse Monoclonal Anti-mouse CD45.2 Alexa Fluor® 700	BioLegend	Cat#109821; Clone 104; RRID: AB_493730
Armenian Hamster Anti-mouse Notch2 APC	BioLegend	Cat#130713; Clone HMN2-35; RRID: AB_2153642
Rat monoclonal Anti-mouse TruStain FcX™ (CD16/32)	BioLegend	Cat#101319; Clone 93; RRID: AB_1574973
Rat monoclonal Anti-mouse CD16/32 Brilliant Violet™ 510	BioLegend	Cat#101333; Clone 93; RRID: AB_2563692
Armenian Hamster Monoclonal Anti-mouse CD16.2 (FcγRIV)	BioLegend	Cat#149602; Clone 9E9; RRID: AB_2565302
Rat Monoclonal Anti-mouse CD3 Biotin	BioLegend	Cat#100243; Clone 17A2; RRID: AB_2563946
Rat Monoclonal Anti-mouse CD19 Biotin	BioLegend	Cat#115503; Clone 6D5; RRID: AB_313638
Rat Monoclonal Anti-mouse/human B220 (CD45R) Biotin	BioLegend	Cat#103203; Clone RA3-6B2; RRID: AB_312988
Rat Monoclonal Anti-mouse Ter119 Biotin	BioLegend	Cat#116203; Clone Ter119; RRID: AB_313704
Rat Monoclonal Anti-mouse Ly6G Biotin	BioLegend	Cat#127603; Clone 1A8; RRID: AB_1186105
Mouse Monoclonal Anti-mouse NK1.1 Biotin	BioLegend	Cat#108703; Clone PK136; RRID: AB_313390
Rat Monoclonal Anti-mouse/human CD11b FITC	BioLegend	Cat#101205; Clone M1/70; RRID: AB_312788
Rat Monoclonal Anti-mouse/human CD11b Brilliant Violet™ 650	BioLegend	Cat#101239; Clone M1/70; RRID: AB_11125575
Rat Monoclonal Anti-mouse/human CD11b Pacific Blue™	BioLegend	Cat#101223; Clone M1/70; RRID: AB_755985
Rat Monoclonal Anti-mouse Ly6C PE-Cy7	BioLegend	Cat#128017; Clone HK1.4; RRID: AB_1732093
Rat Monoclonal Anti-mouse CD45 Brilliant Violet™ 570	BioLegend	Cat#103136; Clone 30-F11; RRID: AB_10898325
Rat Monoclonal Anti-mouse CD45 Alexa Fluor® 700	BioLegend	Cat#103128; Clone 30-F11; RRID: AB_493715
Mouse Monoclonal Anti-mouse CX3CR1 Alexa Fluor® 488	BioLegend	Cat#149022; Clone SA011F11; RRID: AB_2565705
Mouse Monoclonal Anti-mouse CX3CR1 PE	BioLegend	Cat#149005; Clone SA011F11; RRID: AB_2564314
Rat Monoclonal Anti-mouse CD117 APC-Cy7	BioLegend	Cat#105825; Clone 2B8; RRID: AB_1626280
Rat Monoclonal Anti-mouse CD117 PE	BioLegend	Cat#105807; Clone 2B8; RRID: AB_313216
Rat Monoclonal Anti-mouse CD115 PE	BioLegend	Cat#135505; Clone AFS98; RRID: AB_1937254
Rat Monoclonal Anti-mouse CD115 Alexa Fluor® 488	BioLegend	Cat#135511; Clone AFS98; RRID: AB_11218605
Rat Monoclonal Anti-mouse CD115 Brilliant Violet™ 421	BioLegend	Cat#135513; Clone AFS98; RRID: AB_2562667
Rat Monoclonal Anti-mouse CD115 APC	BioLegend	Cat#135509; Clone AFS98; RRID: AB_2085221
Rat Monoclonal Anti-mouse CD34 PE-Dazzle™ 594	BioLegend	Cat#152209; Clone SA376A4; RRID: AB_2734219
Rat Monoclonal Anti-mouse CD135 Brilliant Violet™ 421	BioLegend	Cat#135313; Clone A2F10; RRID: AB_2562338
Rat Monoclonal Anti-mouse CD135 Biotin	BioLegend	Cat#135307; Clone A2F10; RRID: AB_1953266
Rat Monoclonal Anti-mouse CD135 PE	BioLegend	Cat#135305; Clone A2F10; RRID: AB_1877217
Armenian Hamster Monoclonal Anti-mouse CD11c APC	BioLegend	Cat#117310; Clone N418; RRID: AB_313778
Armenian Hamster Monoclonal Anti-mouse CD11c Brilliant Violet™605	BioLegend	Cat#117334; Clone N418; RRID: AB_2562415
Armenian Hamster Monoclonal Anti-mouse CD11c PE	BioLegend	Cat#117307; Clone N418; RRID: AB_313776
Rat Monoclonal Anti-mouse I-A/I-E Brilliant Violet™ 510	BioLegend	Cat#107635; Clone M5/114.15.2; RRID: AB_2561397
Rat Monoclonal Anti-mouse I-A/I-E Alexa Fluor® 700	BioLegend	Cat#107621; Clone M5/114.15.2; RRID: AB_493727
Rat Monoclonal Anti-mouse I-A/I-E PE	BioLegend	Cat#107607; Clone; RRID: AB_313322
Rat Monoclonal Anti-mouse CD43 PerCP-Cy™ 5.5	BD Biosciences	Cat#562865; Clone S7; RRID: AB_2737851
Rat Monoclonal Anti-mouse F4/80 Brilliant Violet™ 650	BioLegend	Cat#123149; Clone BM8; RRID: AB_2564589
Rat Monoclonal Anti-mouse CD90.2 PE	BioLegend	Cat#140307; Clone 53-2.1; RRID: AB_313322
Rat Monoclonal Anti-mouse CD3 PE	BioLegend	Cat#100205; Clone 17A2; RRID: AB_312662
Rat Monoclonal Anti-mouse CD19 PE	BioLegend	Cat#115507; Clone 6D5; RRID: AB_313642
Rat Monoclonal Anti-mouse/human B220 (CD45R) PE	BD Biosciences	Cat#553089; Clone RA3-6B2; RRID: AB_394619
Rat Monoclonal Anti-mouse Ter119 PE	BioLegend	Cat#116208; Clone Ter119; RRID: AB_313708
Rat Monoclonal Anti-mouse Ly6G PE	BioLegend	Cat#127608; Clone 1A8; RRID: AB_1186099
Mouse Monoclonal Anti-mouse NK1.1 PE	BioLegend	Cat#108707; Clone PK136; RRID: AB_313394
Rat Anti-mouse CD49 b PE	BioLegend	Cat#108907; Clone DX5; RRID: AB_313394
Rat Monoclonal Anti-mouse Ly6A/E PE	BioLegend	Cat#108107; Clone D7; RRID: AB_313344
Rat Monoclonal Anti-mouse Ly6A/E Biotin	BioLegend	Cat#108104; Clone D7; RRID: AB_313340
Armenian Hamster Anti-mouse FcεRIα PE	BioLegend	Cat#134307; Clone MAR-1; RRID: AB_1626104
Rat Monoclonal Anti-mouse CD319 PE	BioLegend	Cat#152005; Clone 4G2; RRID: AB_2632677
Rat Monoclonal Anti-mouse CD177 Alexa Fluor® 647	BD Biosciences	Cat#566599; Clone Y127; RRID: AB_2869790
Rat Monoclonal Anti-mouse CD74 Alexa Fluor® 647	BioLegend	Cat#151003; Clone ln1/CD74; RRID: AB_2632608
Mouse Monoclonal Anti-mouse NR4A1 PE	eBioscience	Cat#12-5965-80; Clone 12.14; RRID: AB_1257210
Rat Monoclonal Anti-mouse OCT2 Alexa Fluor® 647	BD Biosciences	Cat#563472; Clone 9A2; RRID: AB_2738229
Rat Monoclonal Anti-mouse CCR2 Alexa Fluor® 700	R&D Systems	Cat#FAB5538N; Clone 475301; RRID: AB_2725739
Streptavidin PE-Dazzle™ 594	BioLegend	Cat#405247; RRID: AB_3697286
Streptavidin PerCP-Cy™ 5.5	BioLegend	Cat#405214; RRID: AB_2716577
Propidium iodide (PI)	Sigma-Aldrich	Cat#81845
4′,6-diamidino-2-phenylindole (DAPI)	Carl-Roth	Cat#6335.1
MojoSort™ Mouse anti-PE beads	BioLegend	Cat#480080
MojoSort™ Streptavidin Nanobeads	BioLegend	Cat#480016

**Chemicals, peptides, and recombinant proteins**		

5-Bromo-2-deoxyuridine (BrdU)	Sigma-Aldrich	Cat#B5002-100 MG
BD Cytoperm™ Permeabilization Buffer Plus	BD Biosciences	Cat#561651
Collagenase Type II	Worthington	Cat#LS004177
Deoxyribonuclease I (DNAse)	Sigma-Aldrich	Cat#D4527
Fraction V, BSA Bovine Serum Albumin (BSA)	Roth	Cat#8076.2
Histopaque®-1083	Sigma-Aldrich	Cat#10831-100 ML
Red Blood Cell Lysis Buffer (10×)	BioLegend	Cat#420301
Mouse M-CSF Recombinant protein PeproTech®	PeproTech	Cat#315-02
Mouse GM-CSF Recombinant protein PeproTech®	PeproTech	Cat#315-03
Recombinant Mouse IgG2A-Fc	R&D Systems	Cat#4460-MG-100
Recombinant Mouse DLL1-Fc Chimera Protein	R&D Systems	Cat#5026-DL
Recombinant Mouse DLL4-Fc Chimera Protein	R&D Systems	Cat#10089-D4

**Critical commercial assays**		

BD Cytofix/Cytoperm™ Kit	BD Biosciences	Cat#554714
BD APC BrdU Kit	BD Biosciences	Cat#552598
SuperScript™ III First-Strand Synthesis System	Invitrogen	Cat#18080051

**Deposited data**		

Raw and analyzed data from scRNA-seq	This paper, GEO	Figures 5, 6 and S7–S14 GEO: GSE289018https://www.ncbi.nlm.nih.gov/geo/query/acc.cgi?acc=GSE289018

**Experimental models: Organisms/strains**		

Mouse: CD45.1^+^	The Jackson Laboratory	Strain#002014; RRID:IMSR_JAX:002014
Mouse: *Cx3cr1*^*Cre*^	Yona et al.^9^ The Jackson Laboratory	Strain#025524; RRID:IMSR_JAX:025524
Mouse: *Cx3cr1*^*GFP/+*^	Jung et al.^43^ The Jackson Laboratory	Strain #:005582 RRID:IMSR_JAX:005582
Mouse: *Cx3cr1*^*Cre*^*Notch2*^*lox/lox*^	This paper	N/A
Mouse: *Cx3cr1*^*Cre*^*Rbpj*^*lox/lox*^	This paper	N/A
Mouse: *Notch2*^*lox/lox*^	Besseyrias et al.^44^	N/A
Mouse: *Rbpj*^*lox/lox*^	Han et al.^45^	N/A
Mouse *Lyz2*^*Cre*^	Clausen et al.^46^	N/A
Mouse: *Cx3cr1*^*GFP/+*^*Lyz2*^*Cre*^*Notch2*^*lox/lox*^	Gamrekelashvili et al.^13^	N/A
Mouse: *Cx3cr1*^*GFP/+*^*Lyz2*^*Cre*^*Rbpj*^*lox/lox*^	Krishnasamy et al.^34^	N/A

**Oligonucleotides**		

QRT-PCR Primers	Eurofins	See method details
Hashtag derived Oligos (HTO)		See method details
TotalSeq-A0301	BioLegend	Cat#155801; RRID: AB_2750032
TotalSeq-A0302	BioLegend	Cat#155803; RRID: AB_2750033
TotalSeq-A0303	BioLegend	Cat#155805; RRID: AB_2750034
TotalSeq-A0304	BioLegend	Cat#155807; RRID: AB_2750035
TotalSeq-A0305	BioLegend	Cat#155809; RRID: AB_2750036
TotalSeq-A0306	BioLegend	Cat#155811; RRID: AB_2750037

**Software and algorithms**		

FlowJo v 10.8.1	FlowJo	https://www.flowjo.com/
Graphpad Prism v 9.3.0	GraphPad	https://www.graphpad.com/
CellRanger v7.1.0	10× Genomics	https://www.10xgenomics.com/
Scrnaseq	Dresden-concept Genome Center	https://github.com/ktrns/scrnaseq/
Seurat v 4.0.0	Hao et al.^47^	https://github.com/satijalab/seurat/
R v 4.0.2	The comprehensive R archive network	https://cran.r-project.org/
Scanpy v 1.9.3	Wolf et al.^48^	https://github.com/scverse/scanpy
Scrublet	Wolock et al.^49^	https://github.com/swolock/scrublet
Harmony	Korsunsky et al.^50^	https://github.com/immunogenomics/harmony
scVelo v 0.3.0	Bergen et al.^51^	https://github.com/theislab/scvelo
Scripts used for scRNA-seq analysis	This paper	https://github.com/sgaedcke/Monocyte_heterogeneity_notch

### Experimental model and subject details

#### Mice

*Cx3cr1*^*GFP/+*^,^43^ *Lyz2*^*Cre*^,^46^ *Notch2*^*lox/lox*^,^44^ *Rbpj*^*lox/lox*^,^45^ *Cx3cr1*^*GFP/+*^*Lyz2*^*Cre*^*Notch2*^*lox/lox*^ (*N2*^*ΔMy*^)^13^ and *Cx3cr1*^*GFP/+*^*Lyz2*^*Cre*^*Rbpj*^*lox/lox*^ (*Rbpj*^*ΔMy*^)^34^ mice have been previously described. *Cx3cr1*^*Cre*^ mice^9^ were crossed with *Notch2*^*lox/lox*^^44^ or *Rbpj*^*lox/lox*^^45^ mice to generate *Cx3cr1*^*Cre*^*Notch2*^*lox/lox*^ (*N2*^*ΔCx3cr1*^) or *Cx3cr1*^*Cre*^*Rbpj*^*lox/lox*^ (*Rbpj*^*ΔCx3cr1*^) mice respectively. B6.SJL*-Ptprc*^*a*^*Pepc*^*b*^*/*BoyJ (CD45.1^+^) mice were purchased from central animal facility of Hannover Medical School (ZTL, MHH). Mice were housed under specific pathogen-free conditions in the central animal facility of Hannover Medical School. Described experiments were performed with age and sex matched littermate controls with approval of the local animal welfare board (LAVES, Lower Saxony, Animal Studies Committee).

### Method details

#### Tissue and cell preparation

For preparation of single cell suspension, mice were sacrificed and BM, PB, spleen, aorta, lung, liver and kidney were collected. Erythrocytes were removed from spleen and blood by red blood cell lysis buffer (BioLegend). Liver and kidney were digested in DMEM medium supplemented with 500U/mL Collagenase II (Worthington). Lung was digested in DMEM medium supplemented with 1U/mL Dispase I (Merck). Liver, kidney and lung were additionally dissociated using GentleMACS tissue dissociator (Miltenyi Biotec) and filtered to remove tissue debris. After extensive washing cells were resuspended in PBS containing 10%FCS and 2 mM EDTA, kept on ice, stained and used for flow cytometry or for sorting.

#### Flow cytometry and cell sorting

Non-specific binding to Fc-receptors was blocked with anti-mouse CD16/CD32 (TruStain fcX) and CD16.2 (both from BioLegend) antibodies in single cell suspensions prepared from spleen, PB or BM. After subsequent washing step cells were labeled with primary and secondary antibodies or streptavidin-fluorochrome conjugates and were used for flow cytometry analysis (LSR-II, BD Biosciences) or sorting (FACSAria, FACSAria Fusion, both from BD Biosciences). Antibodies and fluorochromes used for flow cytometry are described in key resources table. Flow cytometry data were analyzed using FlowJo software (FlowJo LLC). Initially, cells were identified based on FSC and SSC characteristics. After the exclusion of doublets (on the basis of SSC-W, SSC-A), relative frequency of each subpopulation from the live cell gate, or absolute number of each subset (calculated from live cell gate and normalized per mg BM, mg spleen, or μl PB) were determined and are shown in the graphs as mean ± SEM, unless otherwise stated. Unsupervised t-distributed stochastic neighbor embedding (t-SNE) analysis^52^ was performed on live Lin^neg^CD45^+^CD11b^lo/-^ population in concatenated samples using FlowJo.

NR4A1 and OCT2 intranuclear staining was performed after surface staining of single cell suspensions. Cells were fixed and permeabilized using an intranuclear staining kit (BioLegend) and stained with respective antibodies (anti-mouse OCT2-AF647 (BD Biosciences), NR4A1-PE (eBioscience) according to manufacturer’s instructions. Flow cytometry and subsequent analysis was performed using LSR-II Flow cytometry and Flowjo software.

#### *In vitro* cell culture and analysis

96 well flat bottom plates were coated at room temperature for 3 h with IgG-Fc, DLL1-Fc, or DLL4-Fc ligands (all from R&D) reconstituted in PBS. Sorted Lin^neg^CD16/32^hi^CD117^neg^CD135^neg^CD34^neg^CD11c^neg^CD11b^+^Ly6C^hi^CD115^+^ inflammatory Ly6C^hi^ monocytes were cultured in Notch ligand-coated plates in the presence of M-CSF or GM-CSF (10 ng mL^−1^, Peprotech) at 37 °C for 48 h unless otherwise stated. In separate experiments CD74, or CD319 and CD177 were used for the purification of GC-like (CD74^neg^ or CD177^+^) and DC-like (CD74^hi^ or CD319^+^) Ly6C^hi^ inflammatory monocytes. Two days after culture, cells were harvested, stained and analyzed by flow cytometry. Frequency of Ly6C^lo^ monocytes (CD11b^+^Ly6C^lo/−^CD11c^+^CD43^+^), MHCII^+^ monocytes (CD11b^+^Ly6C^lo/−^CD11c^neg^I-A/I-E^+^) or MoDCs (CD11b^+^Ly6C^lo/−^CD11c^+^I-A/I-E^+^) in total live CD11b^+^Ly6C^lo/-^ cells served as an indicator of conversion efficiency and is shown in the graphs. Alternatively, cultured cells were harvested four days later and isolated RNA was used for gene expression analysis.

#### BrdU incorporation analysis

For BrdU incorporation analysis mice were injected with a single dose of BrdU. 24 h after, mice were euthanized and BrdU incorporation was determined using flow cytometry and according to the manufacturer’s instructions (BD Biosciences).

#### Cell hashing and single-cell RNA sequencing

##### Cell hashing and sorting

Single cell suspensions were prepared from the BM or spleen of *N2*^*ΔCx3cr1*^, *Rbpj*^*ΔCx3cr1*^ or control mice consisting of two mice of each genotype. BM Cells were counted and equal numbers were taken from each mouse in separate tubes (one tube per mouse).

A cell hashing-based experimental approach via oligo-tagged antibodies (TotalSeq –A Hashtag derived Oligos (HTO), #A1-#A6, BioLegend) was used for sample multiplexing (Table S5). After initial blocking (anti-mouse CD16/32 BV510 and anti-mouse CD16.2 antibodies) cells were incubated with individual HTOs (#A1-#A6) and with anti-mouse CD115 APC. After an extensive washing step, cells were pooled and stained with a cocktail of anti-mouse Lineage-PE (CD90.2, CD3, CD19, B220, Ly6G, Ter119, NK1.1 Ly6A/E), and anti-PE beads (Mojosort BioLegend). Lineage-PE^+^ cells were depleted using QuadroMACS and LS columns (Miltenyi Biotec). Remaining Lin^neg^ cells were stained with anti-mouse CD11b BV650, CD34 PE-Dazzle594, Ly6C PE-Cy7 and CD117 APC-Cy7 and were sorted using FACSAria Fusion (BD Biosciences). Doublets and DAPI^+^ cells were excluded from sorted populations. Gates were set to collect four subpopulations of myeloid cells (Figure S7A; Table S3). These subpopulations, #1 - #4, were mixed with the ratio: 1: 1: 1.5: 2.75 (Table S3) to have representative subpopulations and loaded on one 10× Genomics lane for downstream processing and scRNA-seq.

Spleens from control, *N2*^*ΔCx3cr1*^ or *Rbpj*^*ΔCx3cr1*^ mice (two mice of each genotype) were cut into small pieces, put in individual tubes (one spleen per tube), and digested in ice-cold DMEM medium supplemented with 2% FCS and 500U/mL Collagenase II (Worthington). The digested mix was filtered through nylon mesh and pelleted, and erythrocytes were removed from cell pellets using RBC-lysis buffer for 30 s.

Cells were counted and equal numbers were taken from each mouse in separate tubes (one tube per mouse). After initial blocking (anti-mouse CD16/32 (TruStain fcX) and anti-mouse CD16.2) cells were incubated with individual HTOs (#A1-#A6) and with anti-mouse F4/80 BV650. After an extensive washing step, cells were pooled, stained with a cocktail of anti-mouse Lineage-PE (CD90.2, CD3, CD19, B220, Ly6G, Ter119, CD49b, NK1.1, CD117, FcεRIα), anti-mouse CD11b FITC, CD11c APC, and Ly6C PE-Cy7, and were sorted using FACSAria Fusion (BD Biosciences). After exclusion of doublets and DAPI^+^CD11b/CD11c^Doubleneg^ cells, Lin^neg^ cells were collected (Figure S7B) and loaded on one 10× Genomics lane for subsequent processing and scRNA-seq.

#### Library preparation and sequencing run

Library preparation for single cell mRNA-Seq analysis was performed according to the Chromium NextGEM Single Cell 3ʹ Reagent Kits v3.1 CellSurfaceProtein User Guide (Manual Part Number CG000206 Rev D; 10× Genomics). According to the protocol a given excess of cells was loaded to the 10× controller in order to reach a target number of 25,000 cells per sample. Fragment length distribution of libraries was monitored using ‘Bioanalyzer High Sensitivity DNA Assay’ (Agilent Technologies). Quantification of libraries was performed by use of the ‘Qubit dsDNA HS Assay Kit’ (ThermoFisher Scientific).

Generated mRNA expression libraries were pooled, denatured with NaOH, and were finally diluted to 1.8pM or 2pM according to the ‘Denature and Dilute Libraries Guide’ (Document # 15048776 v02; Illumina).

1.3 mL of denatured pool (including 1% PhiX) was sequenced on an Illumina NextSeq550 sequencer using one high output flowcell for 75 cycles and 400 million clusters (#20024906; Illumina).

HTO libraries was loaded with a molar proportion corresponding to around 5% of sequencing run capacity.

Sequencing was performed according to the following settings: 28bp as sequence read 1; 56bp as sequence read 2; 8bp as index read 1; no index read 2. In total, two such sequencing runs were performed.

#### Data processing and analysis

The 10× Genomics CellRanger analysis pipeline set (v7.1.0) was used with default parameters. Briefly, BCL files were demultiplexed into FASTQ files by cellranger mkfastq using the respective sample sheet with utilized 10X barcodes. Fastq files of each sample from both sequencing runs were combined. The cellranger count pipeline was used to align read data to the reference genome provided by 10X Genomics (Mus musculus reference dataset refdata-gex-mm10-2020-A), counting aligned reads per gene, and calculating summary statistics. Subsequently, outputs from cellranger count of all samples were aggregated, normalized to the same sequencing depth, and then the feature-barcode matrices was recomputed by cellranger aggr.

Simultaneously, feature barcoding data was processed during the cellranger count step with a feature reference build according to the specification of TotalSeq-A antibodies. The output from cellranger count was demultiplexed to retrieve values for each previously pooled sample. Demultiplexing with hashtag oligos was performed using a Seurat (v.4.0.0)^47^ based workflow developed in a collaboration of Dresden-concept Genome Center (TU Dresden) and Research Core Unit Genomics (Hannover Medical School) (https://github.com/ktrns/scrnaseq). Briefly, the workflow (conducted via R version 4.0.2) log-normalized and clustered the HTO counts, classified cells based on normalised HTO data, removed HTO doublet or negative cells, and generated demultiplexed data.

The demultiplexed scRNA-Seq data was processed separately for BM and spleen with Scanpy (version 1.9.3).^48^ In the preprocessing 196 cells expressing less than 350 genes (5 cells in BM, 191 cells in spleen) and 958 cells (660 in BM, 288 in spleen) were removed with more than 5% mt-genes. 111 doublets (29 in BM, 82 in spleen) were detected and removed using Scrublet.^49^ The data was normalized using Scran and a batch correction was done using Harmony.^50^ The clustering was done using Leiden (resolution = 1) and visualized with UMAP. To annotate and identify clusters the expression of known marker genes for various myeloid cell populations from previous studies were used.^21^^,^^22^^,^^24^^,^^53^ Velocity analysis was calculated using the dynamic mode of scVelo (version 0.3.0).^51^ All used scripts are available under https://github.com/sgaedcke/Monocyte_heterogeneity_notch.

#### Induction of acute systemic inflammation using Aldara

Mice were anesthetized, and the back skin was shaved and depilated using depilating crème. Two days after 50mg/mouse/day Aldara (containing 5% Imiquimod (IMQ), from Meda) or Sham crème were applied on depilated skin for five consecutive days.^54^^,^^55^ Mice were euthanized on day 7 and the liver, lung and kidney were collected for further analysis.

#### Adoptive cell transfer experiments

Lin^neg^CD135^neg^CD11c^neg^CD11b^+^Ly6C^hi^CD115^+^CX_3_CR1-GFP^+^ monocytes were sorted from BM or spleen of CD45.2^+^ donor mice and transferred into CD45.1^+^ recipients intravenously (i.v.). 72h after transfer, spleen and BM were collected, and a single cell suspension was prepared. After blocking of Fc receptors (with anti-mouse CD16/CD32, TruStain fcX and CD16.2, both from BioLegend), cells were labeled with biotin-conjugated antibody cocktail containing anti-CD45.1 and anti-Lin (anti-CD3, CD19, B220, NK1.1, Ly6G, Ter119) antibodies; anti-biotin magnetic beads and positive cells were depleted on LS columns (Miltenyi Biotec) according to the manufacturer’s instructions. Donor-derived CD45.1^neg^Lin^neg^ fraction was collected, stained, and analyzed by flow cytometry. CD45.2^+^CD11b^+^CX_3_CR1-GFP^+^Ly6C^lo/-^F4/80^lo/−^CD11c^+^CD43^+^ Ly6C^lo^ monocytes were quantified in spleen and BM as the relative frequency of total donor-derived CD45.2^+^CD11b^+^CX_3_CR1-GFP^+^ cells.

#### Quantitative real-time PCR analysis

Total RNA was purified from cell lysates using a Nucleospin RNA II kit (Macherey Nagel). After purity and quality check, RNA was transcribed into cDNA employing a cDNA synthesis kit (Invitrogen) according to the manufacturer’s instructions. Quantitative real-time PCR (QRT-PCR) was performed using specific primers for the indicated genes (Table S6) and with FastStart Essential DNA Green Master on a LightCycler 96 system from Roche according to the manufacturer’s instructions. Expression of each specific gene was normalized to expression of *Rps9* and calculated by the comparative CT (2^−ΔCT^) method.^56^

### Quantification and statistical analysis

Statistical analysis was performed using GraphPad Prism software. Details of statistical analysis can be found in the figure legends. Results are expressed as mean ± standard error of the mean (SEM). Significance of differences was calculated using an unpaired, two-tailed Student’s t test with a confidence interval of 95%. For comparison of multiple experimental groups, one-way or two-way ANOVA with Bonferroni’s multiple-comparison test was performed.
