# Supplementary material for: Systematic monocyte subset analysis reveals differential contribution of Notch signaling components to monocyte heterogeneity
Source: iScience. 2025 Oct 16;28(11):113795. doi: 10.1016/j.isci.2025.113795 (PMC12613001; doi:10.1016/j.isci.2025.113795)
Supplement: Document S1. Figures S1–S16 and Tables S1–S6 [file mmc1.pdf]

iScience, Volume *28*

Supplemental information

Systematic monocyte subset analysis reveals differential contribution of Notch signaling components to monocyte heterogeneity

Yuangao Xu, Tamar Kapanadze, Svenja Gaedcke, Adan Chari Jirmo, Stefan Sablotny, Frauline Nicole Schroth, Susanne Hille, Oliver J. Müller, Matthias Lochner, Hermann Haller, Kai Schmidt-Ott, Jaba Gamrekelashvili, and Florian P. Limbourg

Supplementary Figures

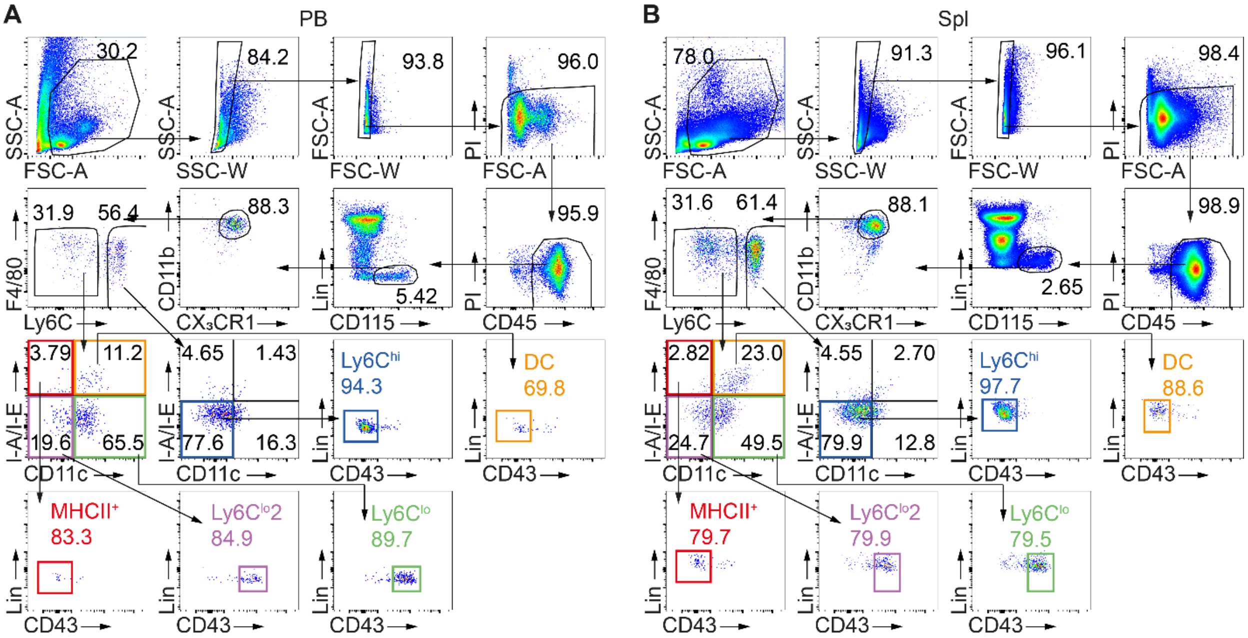

Figure S1. Flow cytometry gating strategy of mononuclear phagocytes, related to Figure 2. (**A, B**) Representative gating strategy for definition, quantitative and qualitative analysis of monocyte subsets and DC by flow cytometry in PB (**A**) or Spleen (**B**). Monocyte subsets were defined from the Lin^{neg}CD45^{+}CD115^{+}CD11b^{+} gate after excluding doublets and dead Propidium Iodide^{+} (PI^{+}) cells.

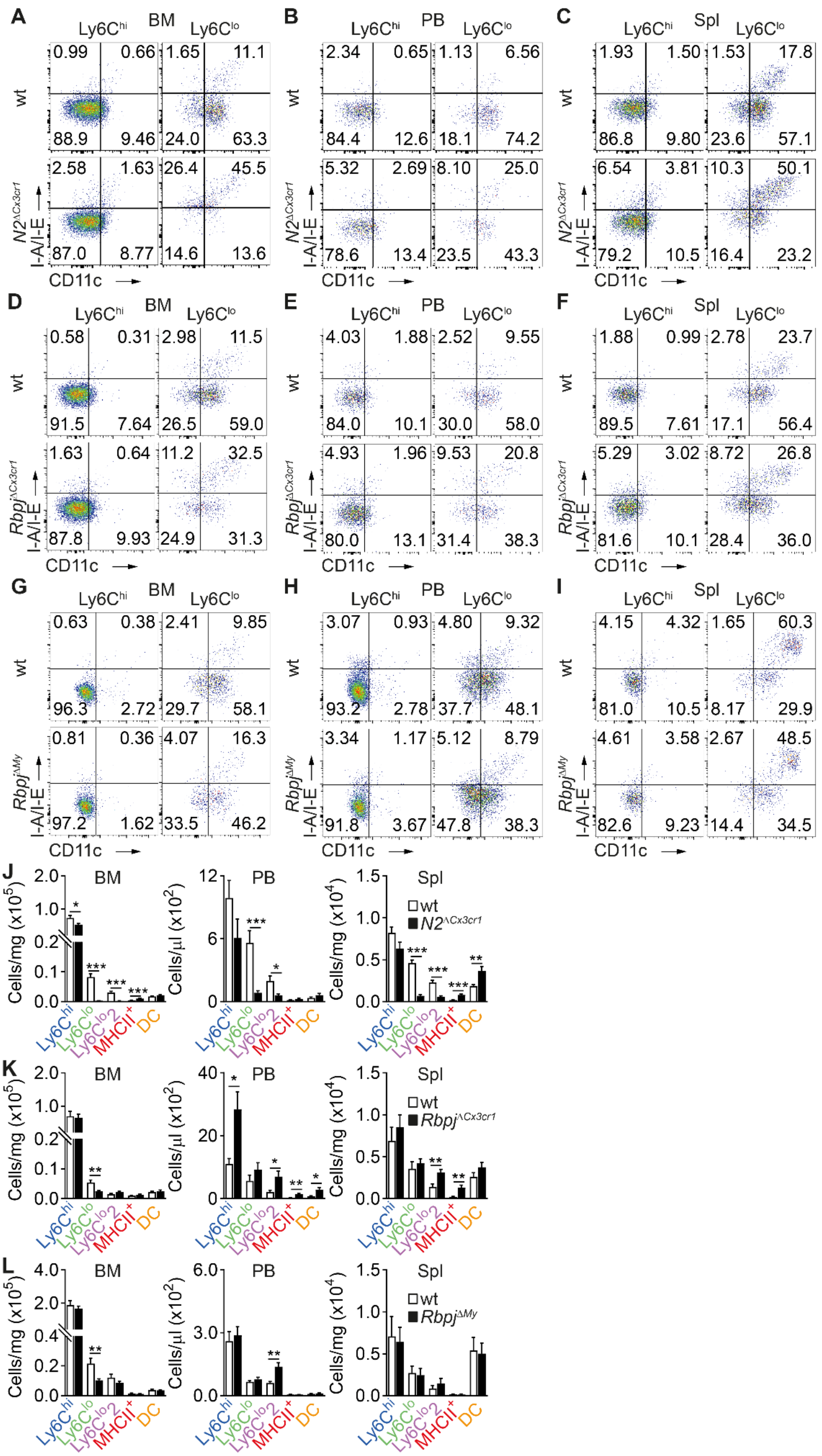

Figure S2. Flow cytometry analysis of myeloid cell subsets in Notch2 and Rbpj knock-out mice, related to Figure 2.

(**A-I**) Representative two-dimensional pseudocolor plots showing expression of CD11c and I-A/I-E on live $Lin^{neg}CD45^{+}CD115^{+}CD11b^{+}Ly6C^{hi}F4/80^{lo/-}$ and $Lin^{neg}CD45^{+}CD115^{+}CD11b^{+}Ly6C^{lo/-}F4/80^{lo/-}$ subsets from

BM (**A, D, G**), PB (**B, E, H**), and Spl (**C, F, I**) of *N2*$^{\Delta Cx3cr1}$ (**A-C**), *Rbpj*$^{\Delta Cx3cr1}$ (**D-F**), and *Rbpj*$^{\Delta My}$ (**G-I**) mice. *Cre*-negative littermates (wt) were used as controls.
(**J-L**) Absolute numbers of different monocyte subpopulations in BM, PB and Spl of *N2*$^{\Delta Cx3cr1}$- (**J**), *Rbpj*$^{\Delta Cx3cr1}$- (**K**), or *Rbpj*$^{\Delta My}$ (**L**) mice, and appropriate littermate controls (wt) are shown as mean ± SEM.
(**J-L**) Data are pooled from two experiments, with n=8 (**J**) and n=6-8 (**K, L**) mice. (**J-L**) * p<0.05, ** p<0.01, *** p<0.001; Two-tailed unpaired Student's t-test.

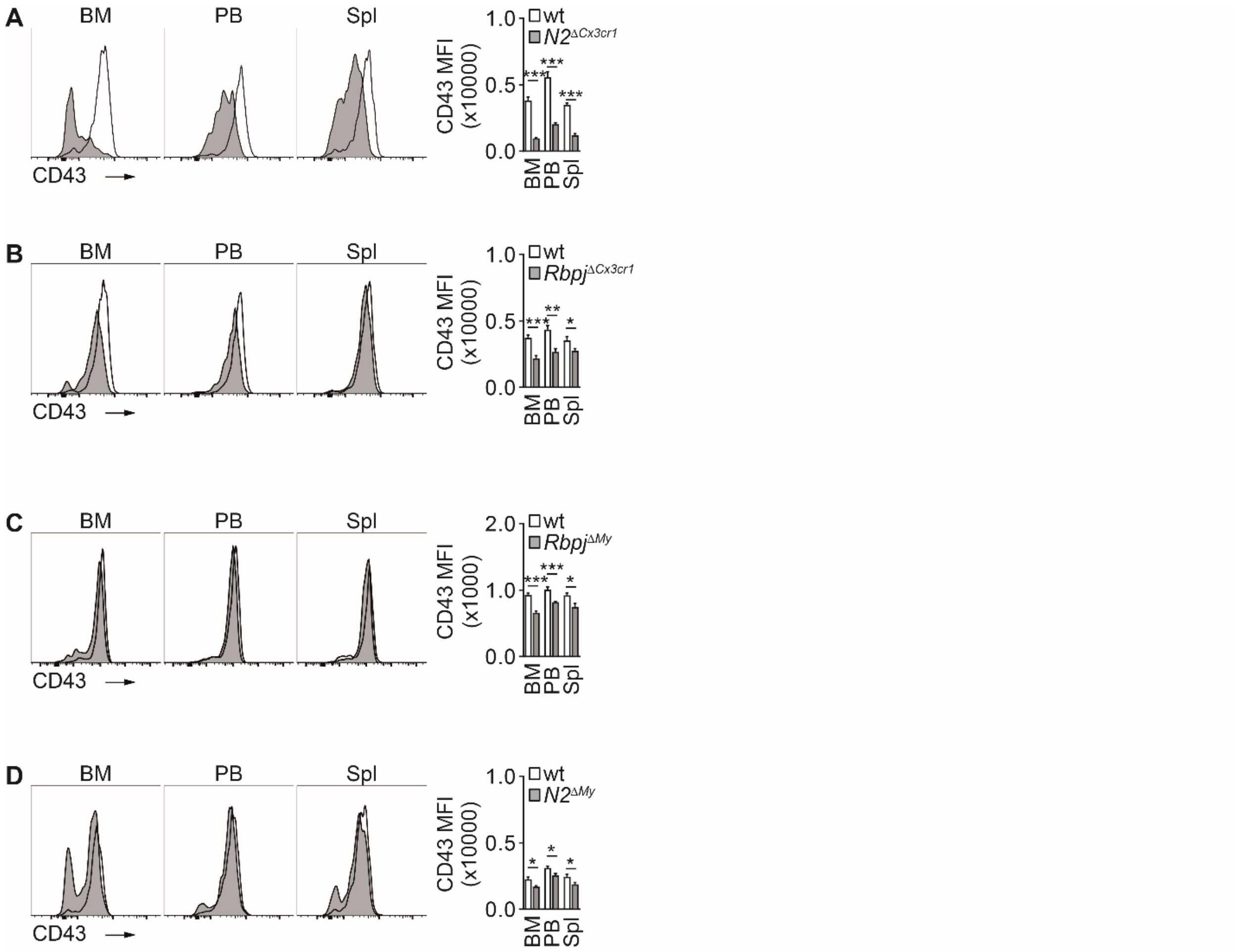

Figure S3. Notch2- and Rbpj-dependent differences in monocyte phenotype, related to Figure 3.
(**A-D**) Expression of CD43 on live $Lin^{neg}CD45^{+}CD115^{+}CD11b^{+}Ly6C^{lo/-}F4/80^{lo/-}CD11c^{lo}I\text{-}A/I\text{-}E^{lo/-}$ ($Ly6C^{lo}$) monocytes from BM, PB and Spl of *N2*$^{\Delta Cx3cr1}$ (**A**), *Rbpj*$^{\Delta Cx3cr1}$ (**B**), *Rbpj*$^{\Delta My}$ (**C**), or *N2*$^{\Delta My}$ (**D**) and wt control mice analyzed by flow cytometry. Representative histogram plots and bar graphs with MFI of CD43 are shown. Data are representative of three experiments (**A**), or pooled from two (**B, C**) or three (**D**) experiments; n=4 (**A**), n=7/6 (**B**), n=6/8 (**C**), n=9/8 (**D**).
(**A-D**) Data are shown as mean ± SEM; * p<0.05, ** p<0.01, *** p<0.001; Student's *t*-test.

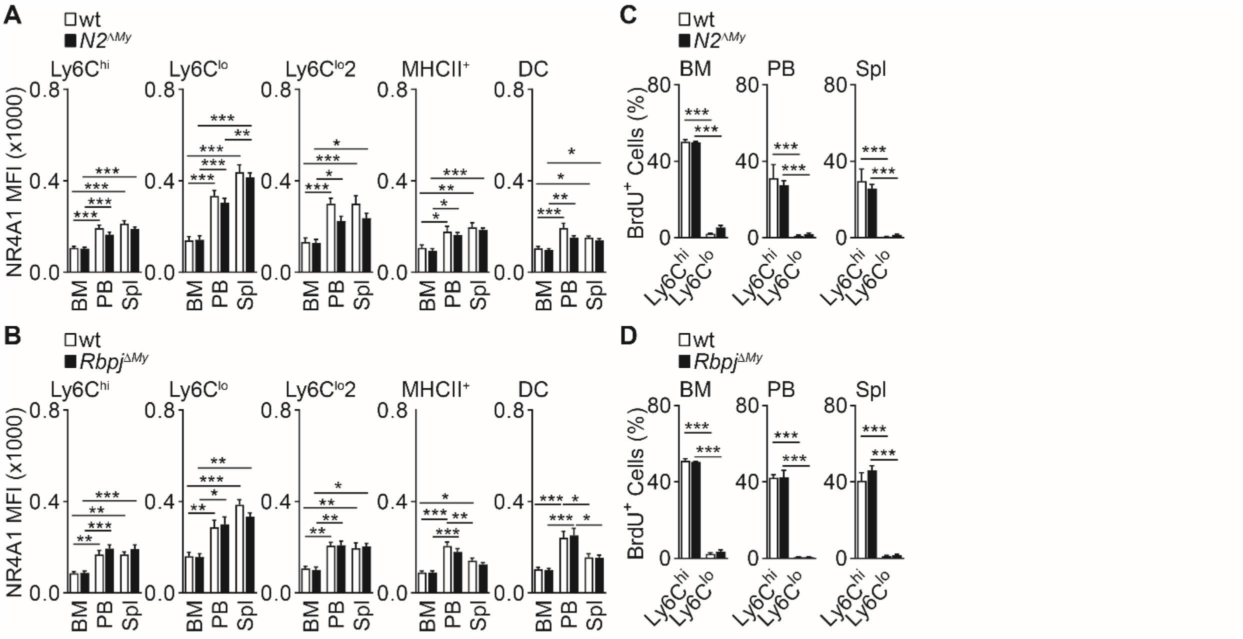

Figure S4. Notch2 and Rbpj differently regulate monocyte cell fate, related to Figure 3.
(**A, B**) Expression of NR4A1 in Ly6C^{hi}, Ly6C^{lo}, Ly6C^{lo}2, and MHCII^{+} monocytes and DCs from BM, PB and Spl of *N2*$^{\Delta My}$ - (**A**), or *Rbpj*$^{\Delta My}$ (**B**) mice analyzed by flow cytometry after intranuclear staining. Bar graphs showing the MFI of NR4A1. Data are pooled from two experiments; n=10 (**A**), n=6/4 (**B**).
(**C, D**) Proliferation potential of monocyte subsets in *N2*$^{\Delta My}$ - (**C**), or *Rbpj*$^{\Delta My}$ (**D**) and wt control mice analyzed by BrdU incorporation and flow cytometry. Bar graphs showing the relative frequency of BrdU^{+} cells. Data are representative of two experiments; n=4/6 (**C**), n=3/4 (**D**).
(**A-D**) Data are shown as mean ± SEM; * p<0.05, ** p<0.01, *** p<0.001; two-way ANOVA with Bonferroni's multiple comparison test.

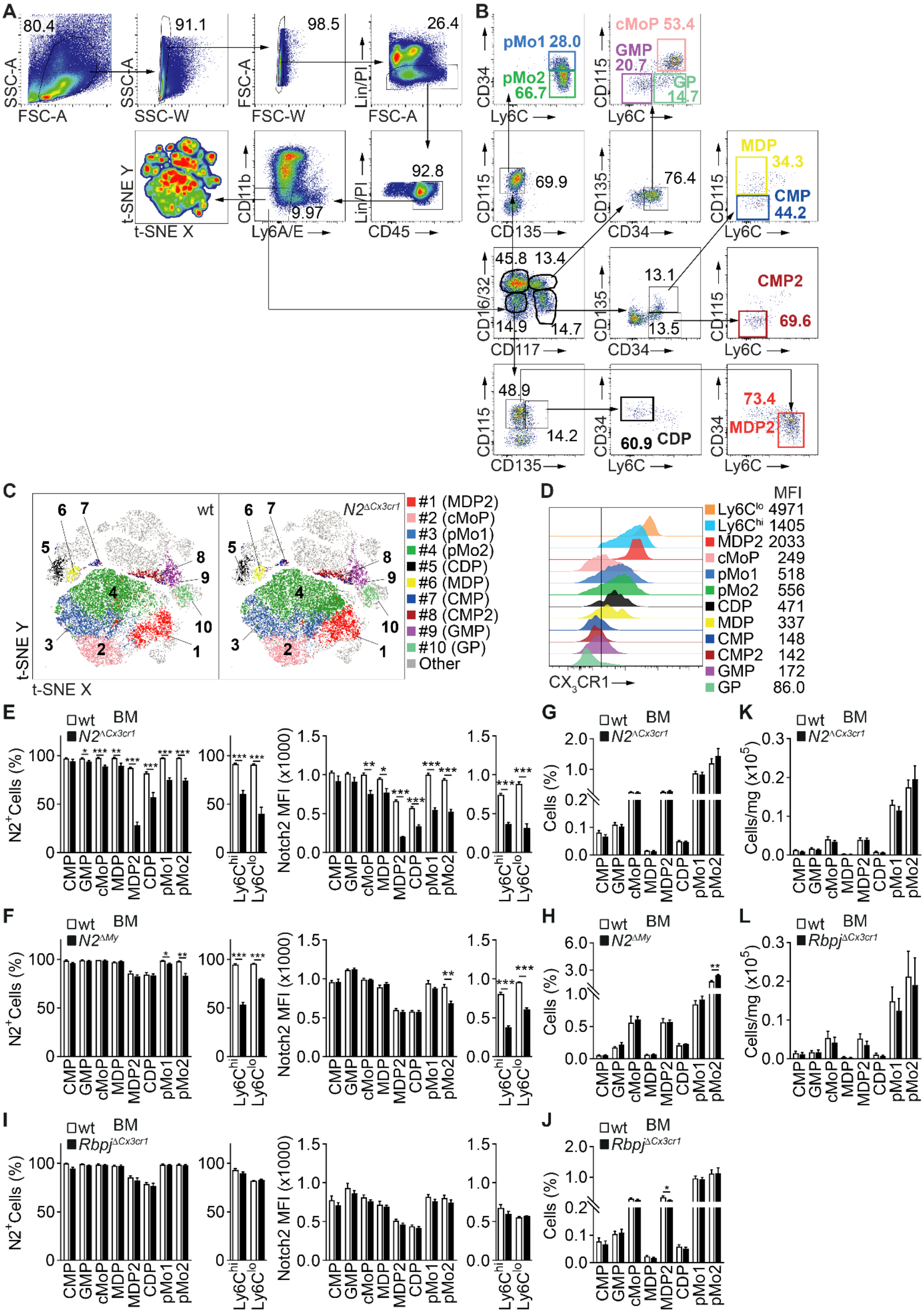

Figure S5. Myeloid lineage in Notch2- and Rbpj-deficient bone marrow.
(**A, B**) Gating strategy for unsupervised t-SNE (**A**) and for conventional flow cytometry (**B**) analysis. (**A**) t-SNE analysis was performed on concatenated live Lin^{neg}CD45^{+}CD11b$^{lo/neg}$ cells isolated from BM of wt control or *N2*$^{\Delta Cx3cr1}$ mice (3 mouse each). (**B**) The same live Lin^{neg}CD45^{+}CD11b$^{lo/neg}$ cells were used for conventional flow cytometry gating strategy-based analysis of myeloid progenitors in the BM.

(**C**) Unsupervised t-SNE analysis and definition of cell subsets based on expression of specific cell surface markers. t-SNE was performed on live $Lin^{neg}CD45^{+}CD11b^{lo/neg}Ly6A/E^{neg}$ BM cells concatenated from $N2^{\Delta Cx3cr1}$ and control (wt) mice (n=6).
(**D**) Representative flow cytometry histogram showing expression and MFI of CX_3CR1-GFP in different myeloid subsets as a potential indicator for efficiency of $Cx3cr1^{Cre}$-mediated targeting strategy.
(**E**) Frequency of $Notch2^{+}$ cells and expression of Notch2 in different myeloid subsets from the BM of $N2^{\Delta Cx3cr1}$ and control mice. Bar graphs depicting the relative frequency of $Notch2^{+}$ cells, or mean fluorescence intensity (MFI) of Notch2. Data are pooled from two experiments (n=7/6).
(**F**) Frequency of $Notch2^{+}$ cells and expression of Notch2 in different myeloid subsets from the BM of $N2^{\Delta My}$ and control mice. Bar graphs depicting the relative frequency of $Notch2^{+}$ cells, or mean fluorescence intensity (MFI) of Notch2. Representative of two experiments (n=3/4).
(**G**) Relative frequency of different myeloid subsets in the BM of $N2^{\Delta Cx3cr1}$ and control mice. Data are pooled from two experiments (n=7/6).
(**H**) Relative frequency of different myeloid subsets in the BM of $N2^{\Delta My}$ and control mice. Representative of two experiments are shown (n=3/4).
(**I**) Frequency of $Notch2^{+}$ cells and expression of Notch2 in different myeloid subsets from the BM of $Rbpj^{\Delta Cx3cr1}$ and control mice. Bar graphs depicting the relative frequency of $Notch2^{+}$ cells, or mean fluorescence intensity (MFI) of Notch2. Pooled from two experiments (n=7/8).
(**J**) Relative frequency of different myeloid subsets in the BM of $Rbpj^{\Delta Cx3cr1}$ and control mice are shown. Data are pooled from two experiments (n=7/8).
(**K**) Absolute frequency of different myeloid subsets in the BM of $N2^{\Delta Cx3cr1}$ and control mice. Data are pooled from two experiments (n=7/6).
(**L**) Absolute frequency of different myeloid subsets in the BM of $Rbpj^{\Delta Cx3cr1}$ and control mice are shown. Data are pooled from two experiments (n=7/8).
(**E-L**) Data are shown as mean ± SEM;* $p<0.05$, ** $p<0.01$, *** $p<0.001$; unpaired Student's *t*-test.

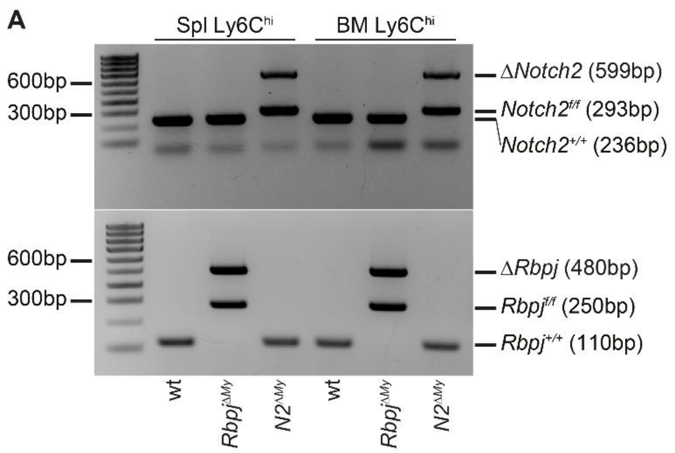

Figure S6. *Cre*-mediated strategy of deletion targets *Notch2* and *Rbpj* loci in Ly6C^{hi} inflammatory monocytes.
(**A**) Representative PCR for wt, floxed or recombined locus of *Notch2* (*ΔNotch2*) and *Rbpj* (*ΔRbpj*) in Ly6C^{hi} monocytes sorted from BM and Spl of wt control, *Rbpj*$^{\Delta My}$ and *Notch2*$^{\Delta My}$ mice.

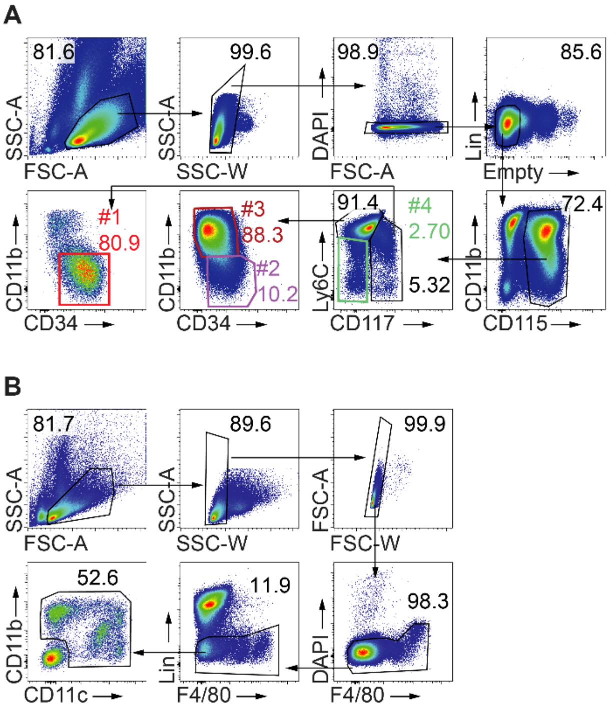

Figure S7. Sorting strategy of BM and spleen cells for scRNA-seq analysis, related to Figures 5 and 6.

(**A**) Gating strategy for sorting of myeloid subsets from $N2^{\Delta Cx3cr1}$, $Rbpj^{\Delta Cx3cr1}$, or control BM for scRNA-seq analysis.

(**B**) Gating strategy for sorting of myeloid subsets from spleens of $N2^{\Delta Cx3cr1}$, $Rbpj^{\Delta Cx3cr1}$, or control mice for scRNA-seq analysis

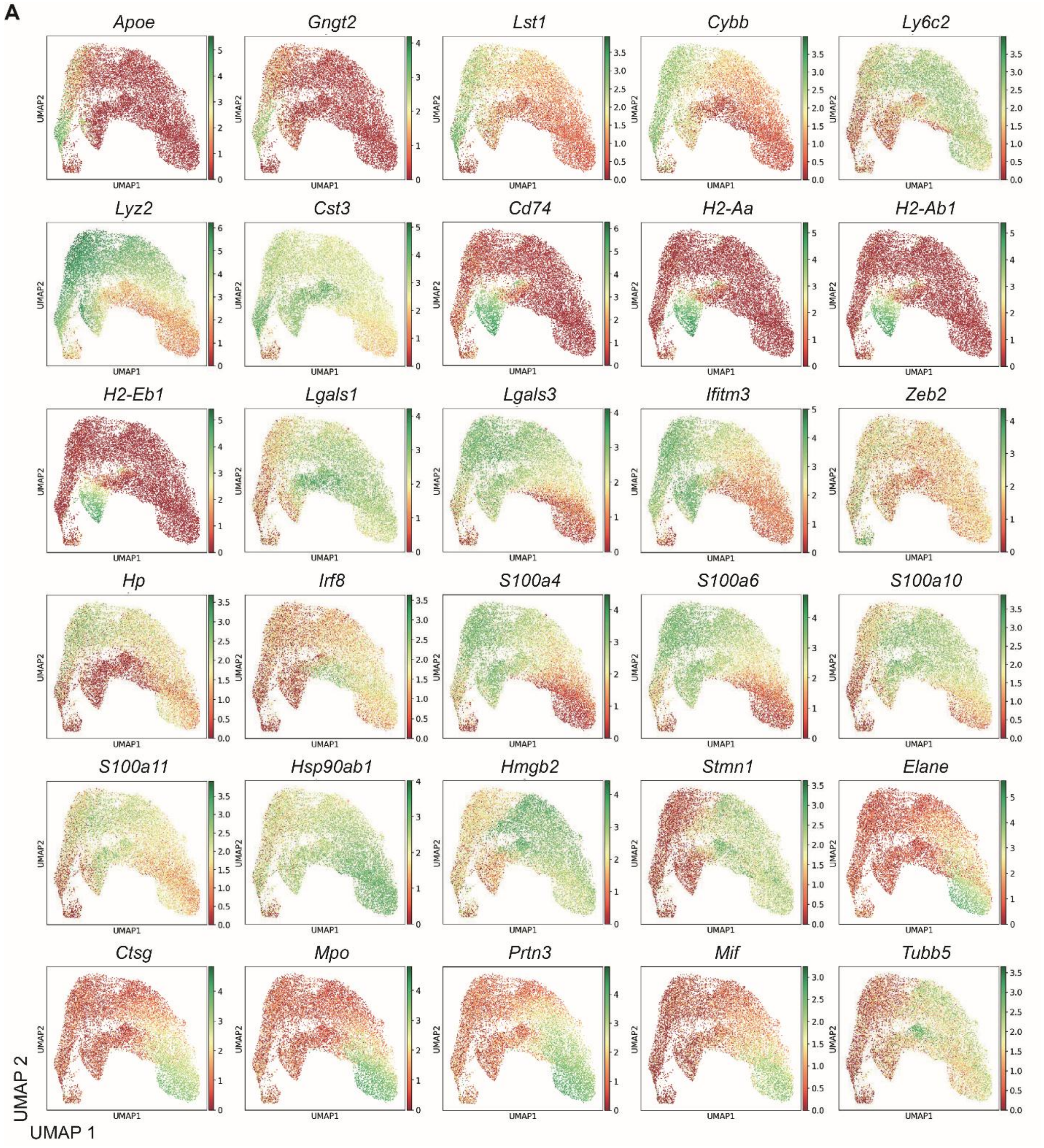

Figure S8. Expression of selected genes in BM scRNA-seq data, related to Figure 5.
(**A**) Two-dimensional UMAP plots of BM cells multiplexed from *N2*$^{\Delta Cx3cr1}$, *Rbpj*$^{\Delta Cx3cr1}$, or control mice showing expression of selected genes and used for mapping of myeloid subsets.

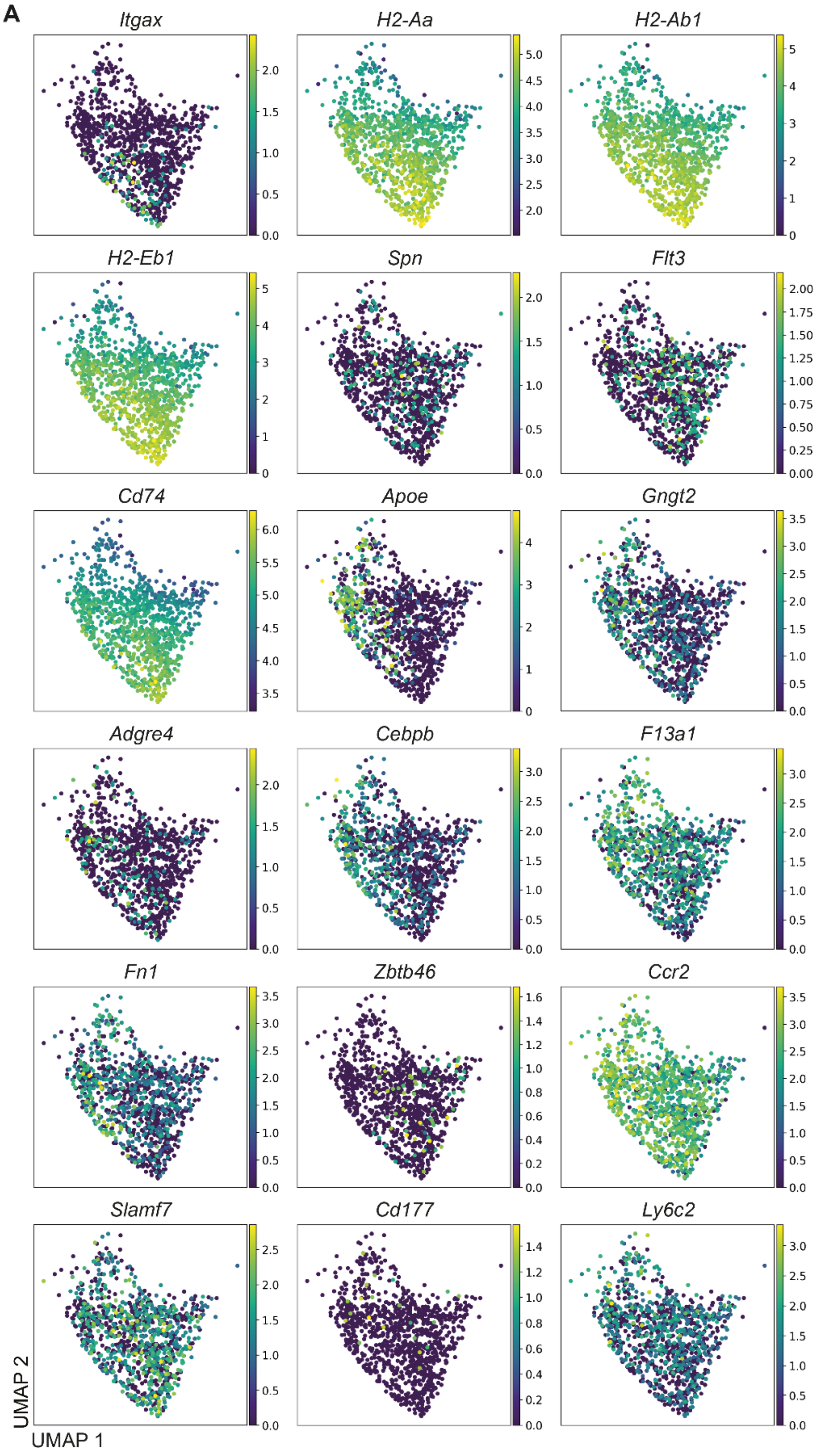

Figure S9. Expression of selected genes in monocyte cluster #4 from BM scRNA-seq data, related to Figure 5.

(**A**) Two-dimensional UMAP plots of BM cluster #6 (MHCII^{+} monocytes) multiplexed from *N2*$^{\Delta Cx3cr1}$, *Rbpj*$^{\Delta Cx3cr1}$, or control mice showing expression of selected genes typical for monocytes or DCs.

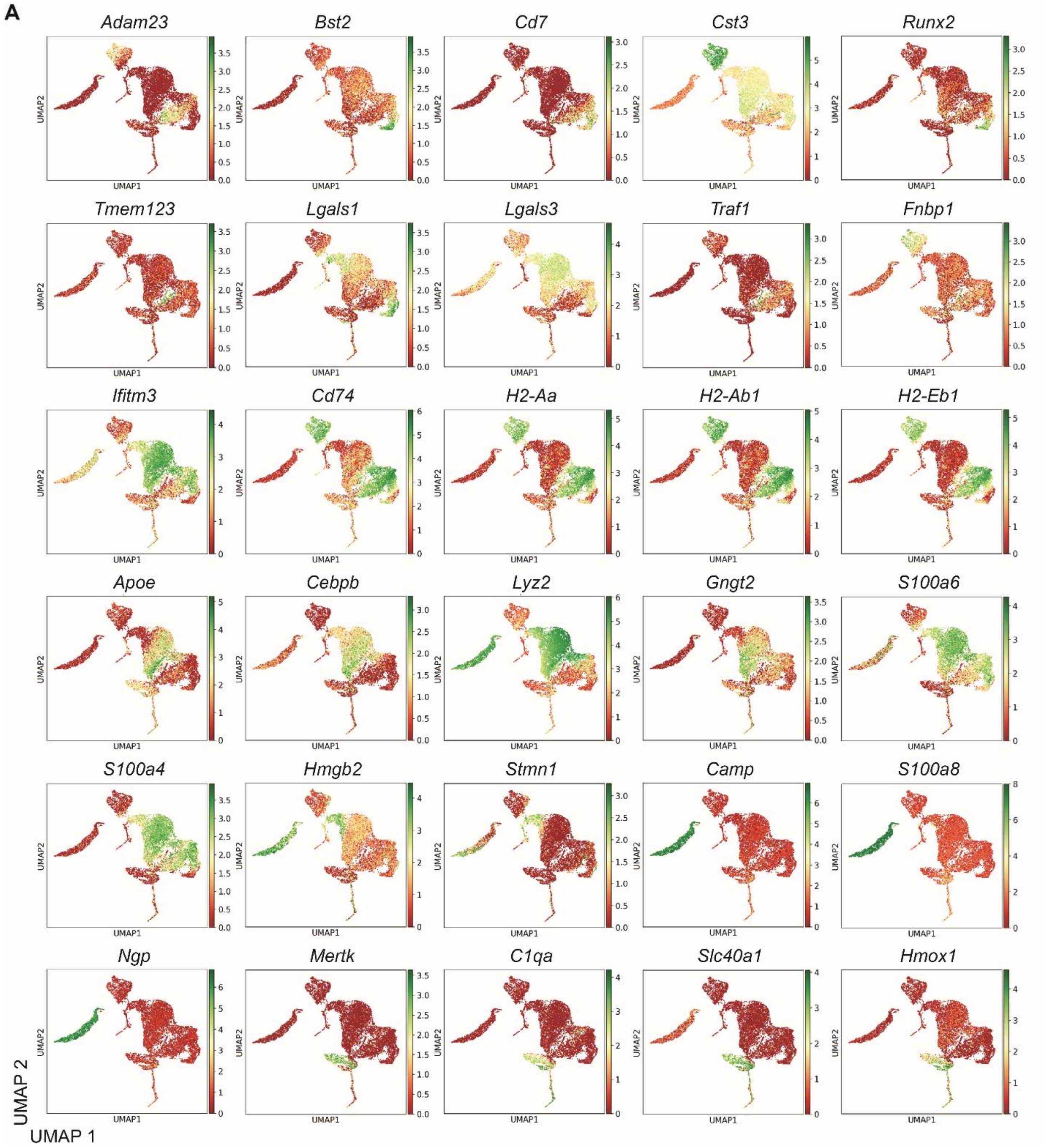

Figure S10. Expression of selected genes in spleen scRNA-seq data, related to Figure 6.
(**A**) Two-dimensional UMAP plots of sorted Spl cells multiplexed from *N2*$^{\Delta Cx3cr1}$, *Rbpj*$^{\Delta Cx3cr1}$, or control mice showing expression of selected genes and used for mapping of cellular subsets.

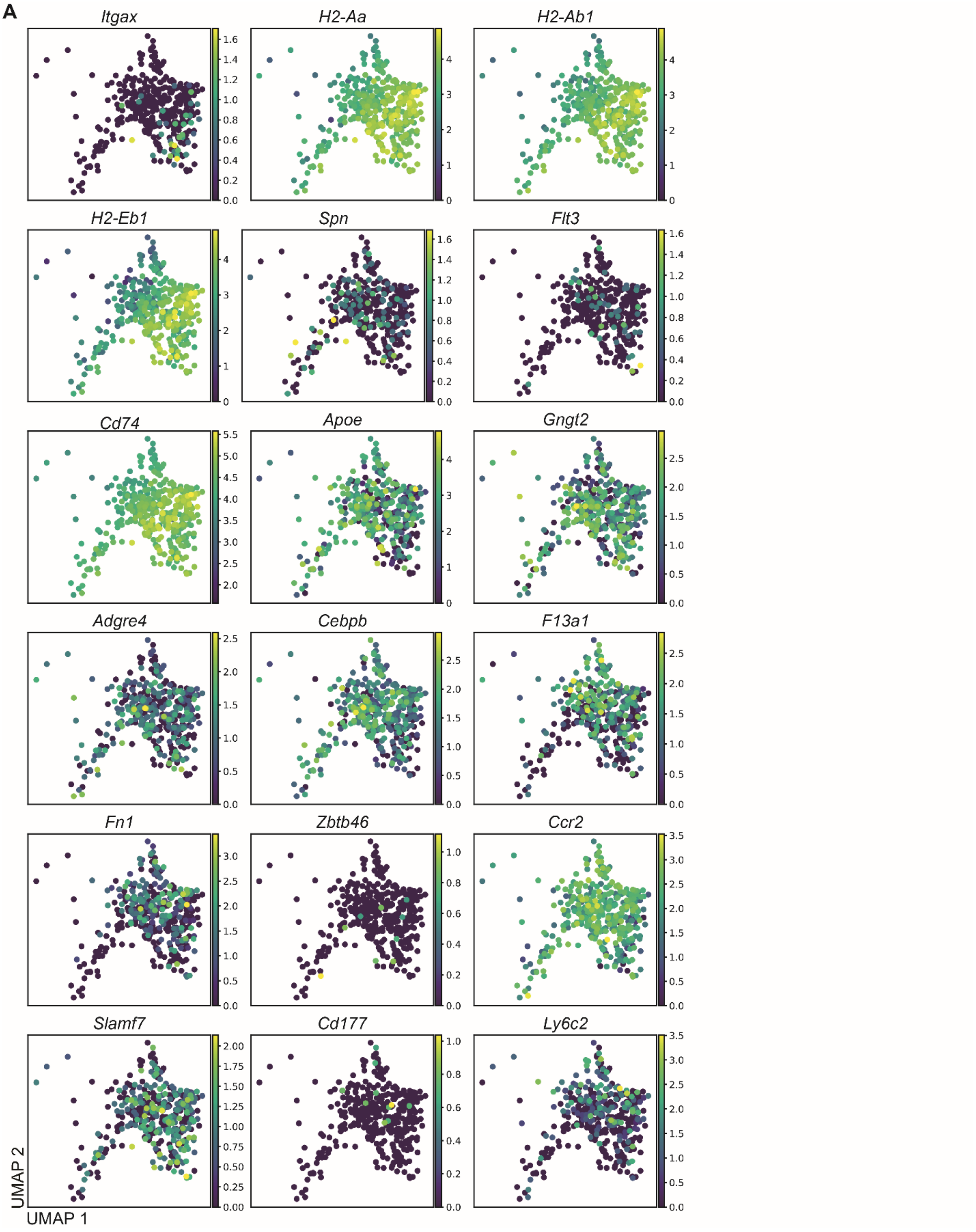

Figure S11. Expression of selected genes in MHCII^{+} monocyte cluster #5 from spleen scRNA-seq data, related to Figure 6.
(**A**) Two-dimensional UMAPs of Spl monocyte cluster #8 (MHCII^{+}) multiplexed from *N2*$^{\Delta Cx3cr1}$, *Rbpj*$^{\Delta Cx3cr1}$, or control mice showing expression of selected genes typical for monocytes or dendritic cells.

A

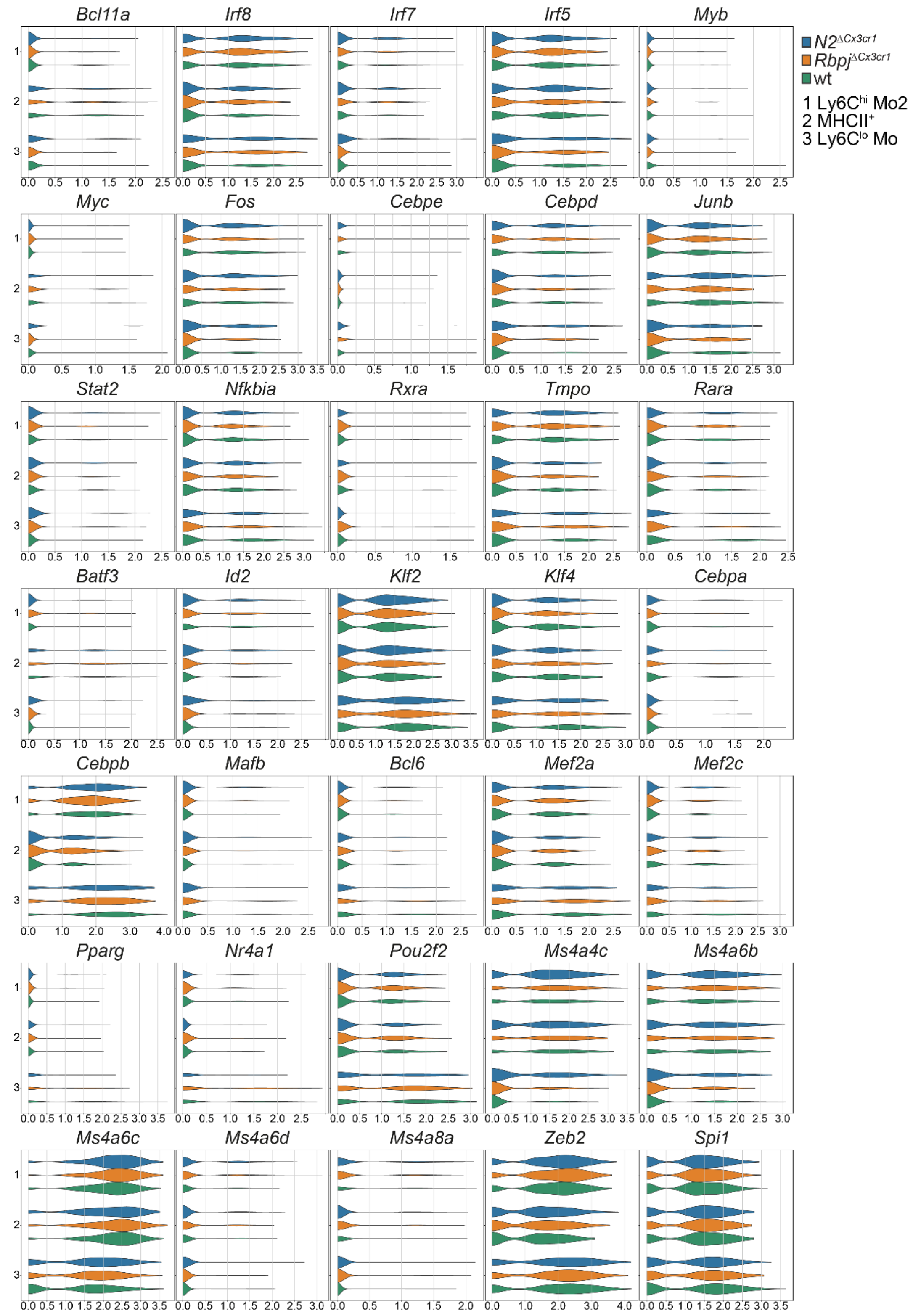

Figure S12. Expression of key monocyte-related transcription factors in monocyte subsets from BM scRNA-seq data, related to Figure 5.

(**A**) Violin plots showing expression of selected transcription factors in monocytes ((1) Ly6C^{hi} Mo2, (2) MHCII^{+}, and (3) Ly6C^{lo} Mo subsets defined in **Figure 5B**) from BM of *N2*$^{\Delta Cx3cr1}$, *Rbpj*$^{\Delta Cx3cr1}$, or control mice.

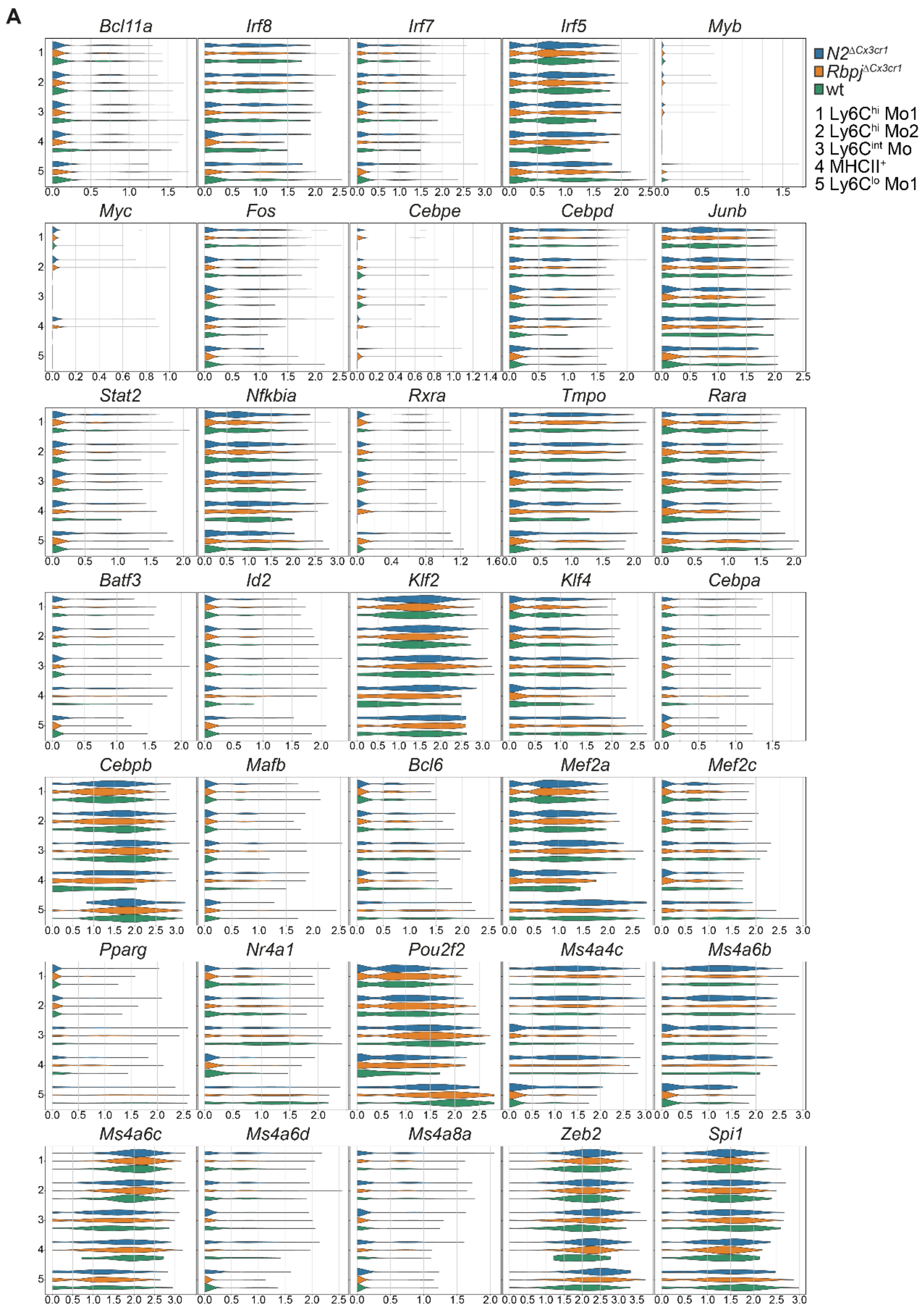

Figure S13. Expression of key monocyte-related transcription factors in monocyte subsets from spleen scRNA-seq data, related to Figure 6.

(**A**) Violin plots showing expression of selected transcription factors in monocyte subsets ((1) Ly6C^{hi} Mo1, (2) Ly6C^{hi} Mo2, (3) Ly6C^{int} Mo, (4) MHCII^{+}, and (5) Ly6C^{lo} Mo1 subsets defined in **Figure 6B**) from spleen of *N2*$^{\Delta Cx3cr1}$, *Rbpj*$^{\Delta Cx3cr1}$, or control mice.

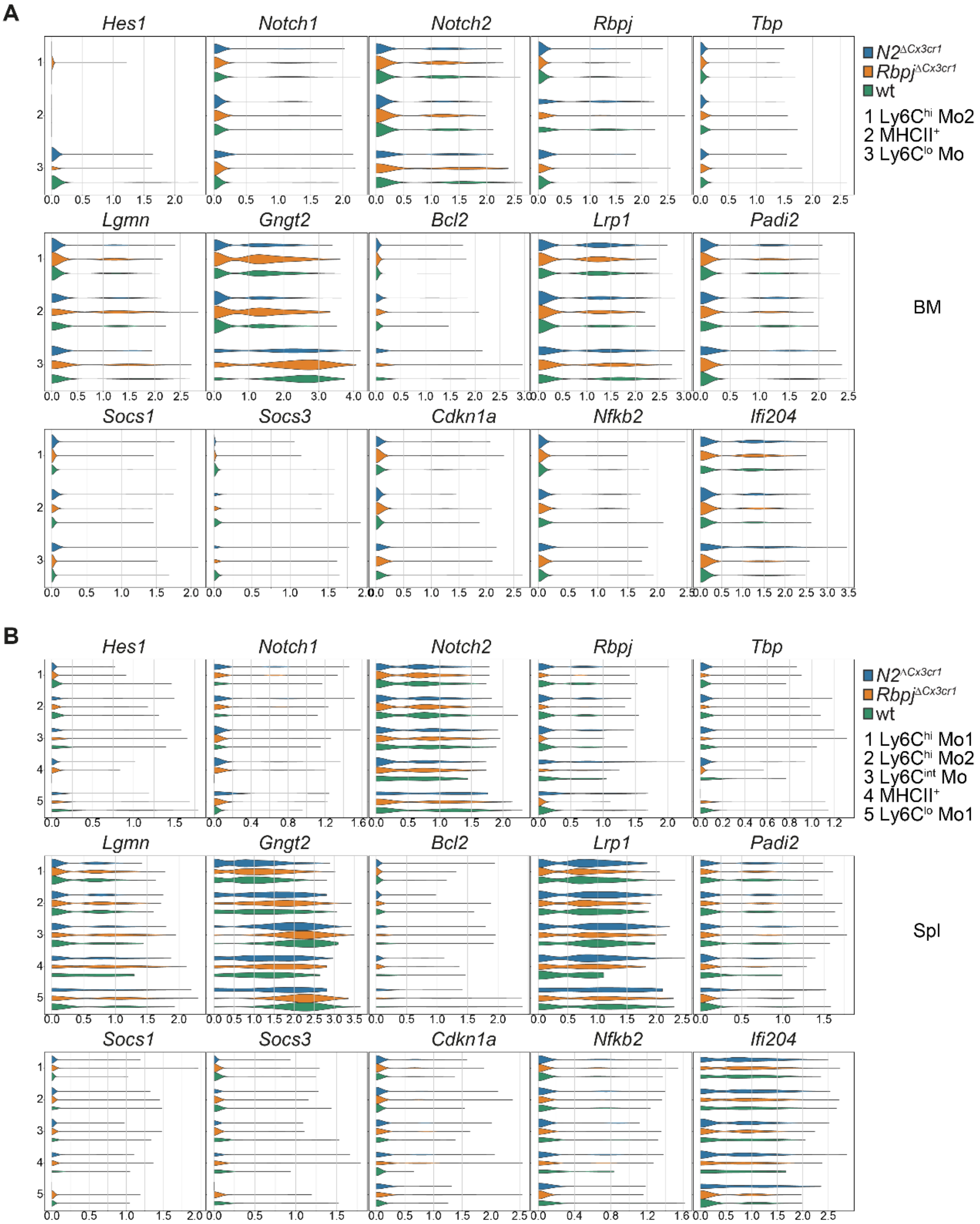

Figure S14. Expression of key Notch-signaling related genes in monocyte subsets from BM and spleen scRNA-seq data, related to Figures 5 and 6.

(**A**) Violin plots showing expression of Notch-signaling related genes in monocytes ((1) Ly6C^{hi} Mo2, (2) MHCII^{+}, and (3) Ly6C^{lo} Mo subsets defined in **Figure 5B**) from BM of *N2*$^{\Delta Cx3cr1}$, *Rbpj*$^{\Delta Cx3cr1}$ or control mice. (**B**) Violin plots showing expression of Notch-signaling related genes in monocyte subsets ((1) Ly6C^{hi} Mo1, (2) Ly6C^{hi} Mo2, (3) Ly6C^{int} Mo, (4) MHCII^{+}, and (5) Ly6C^{lo} Mo1 subsets defined in **Figure 6B**) from spleen of *N2*$^{\Delta Cx3cr1}$, *Rbpj*$^{\Delta Cx3cr1}$, or control mice.

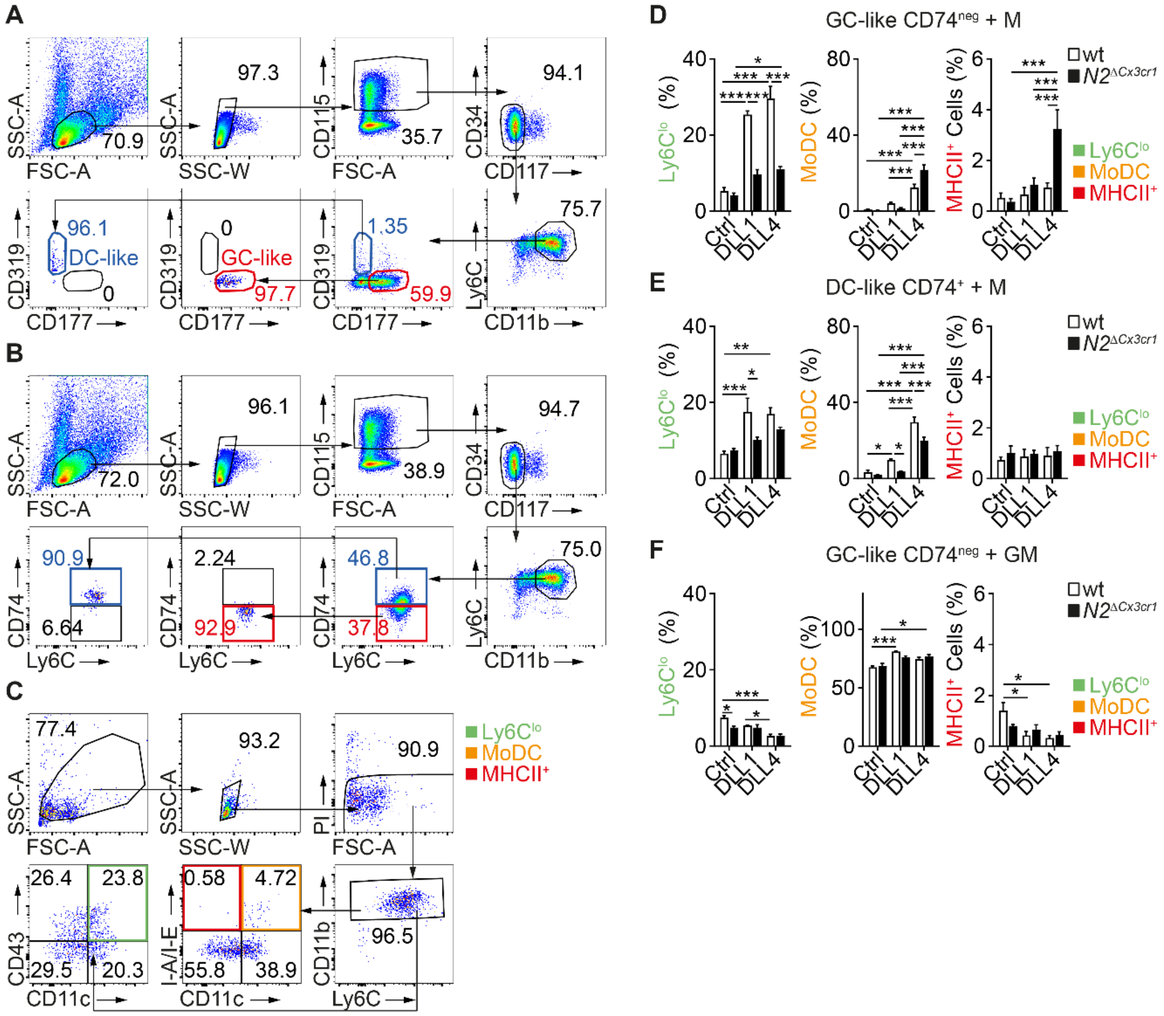

Figure S15. Notch2 controls GC-like and DC-like Ly6C^{hi} monocyte conversion *in vitro*, related to Figure 7.

(**A**) Sorting strategy of CD177^{+}CD319^{neg} GC-like (red gate) and CD177^{neg}CD319^{+} DC-like (blue gate) BM Ly6C^{hi} monocytes for *in vitro* conversion studies.

(**B**) Sorting strategy of CD74^{neg} GC-like (red gate) and CD74^{+} DC-like (blue gate) BM Ly6C^{hi} monocytes for *in vitro* conversion studies.

(**C**) Flow cytomtry gating strategy for analysis of Ly6C^{lo}- (green) and MHCII^{+} (red) monocytes, and Mo-DCs (orange) developed from GC-like and DC-like BM Ly6^{hi} monocytes *in vitro*.

(**D, E**) GC-like (**D**), and DC-like (**E**) Ly6C^{hi} monocytes were sorted from wt or *N2*$^{\Delta Cx3cr1}$ BM and cultured *in vitro*, in the presence of Ctrl, DLL1 or DLL4 ligands and M-CSF (M). The frequency of Ly6C^{lo} monocytes, MHCII^{+} monocytes, and MoDCs were determined using flow cytometry. Data are shown as mean ± SEM; (**D, E**) pooled from three experiments (n=5-9).

(**F**) GC-like Ly6C^{hi} monocytes differentiate into MoDC *in vitro* under the influence of GM-CSF (GM). GC-like CD74^{neg} Ly6C^{hi} monocytes, sorted from wt or *N2*$^{\Delta Cx3cr1}$ BM, were cultured *in vitro* in the presence of Ctrl, DLL1 or DLL4 ligands and GM-CSF. The frequency of Ly6C^{lo} monocytes, MHCII^{+} monocytes, and MoDCs were determined using flow cytometry and are shown as mean ± SEM. Representative of two experiments (n=3).

(**D-F**) * p<0.05, ** p<0.01, *** p<0.001; two-way ANOVA with Bonferroni's multiple comparison test.

A

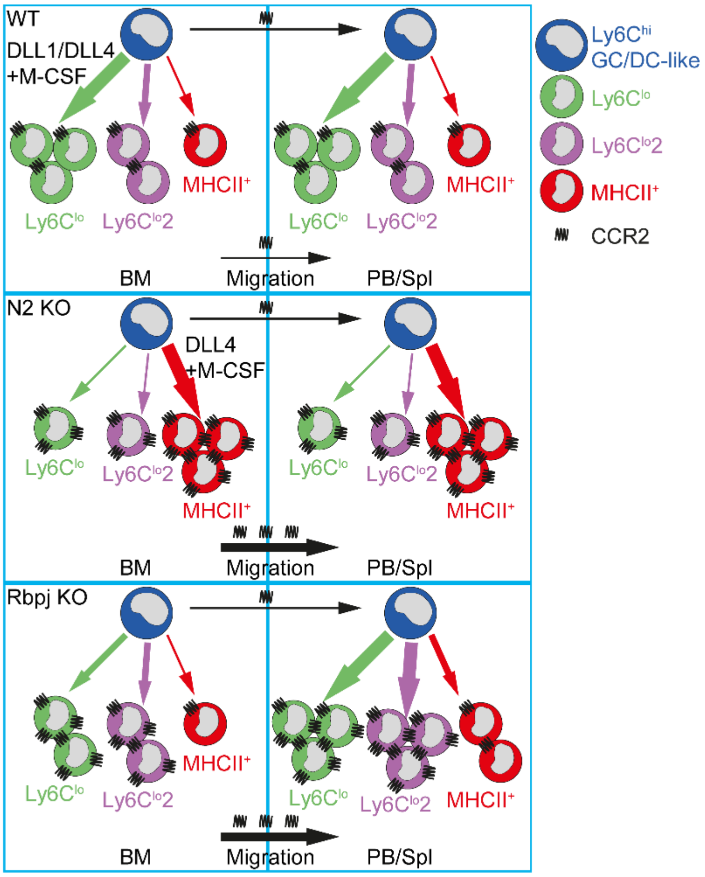

Figure S16. Compartment-specific heterogeneity of monocytes is controlled by Notch signaling.
(**A**) Under steady-state conditions (top row, WT), Ly6C^{hi} inflammatory monocytes tend to convert towards patrolling Ly6C^{lo} and Ly6C^{lo}2 monocytes, which express no or low levels of CCR2 and are 'evenly' distributed in both BM and periphery (PB/Spl). This conversion process is regulated by Notch ligands, DLL1 and DLL4, in the presence of M-CSF *in vitro*.
In the absence of functional Notch2 signaling (middle row, N2 KO), Ly6C^{hi} to Ly6C^{lo}/Ly6C^{lo}2 conversion process is diminished, and MHCII^{+} monocytes (Ly6C^{lo}CD11c^{neg}CD43^{neg}MHCII^{+}) expand. All these cells upregulate (express) abnormally high levels of CCR2, which might lead to enhanced migration from the BM towards the periphery. Development of MHCII^{+} monocytes from Notch2-deficient Ly6C^{hi} monocytes depends on DLL4 and M-CSF signaling *in vitro*.
In the absence of Rbpj signaling (bottom row, Rbpj KO), Ly6C^{hi}-to-Ly6C^{lo} monocyte conversion is not impaired, however, CD11c^{neg} Ly6C^{lo}2 monocytes expand. Both Ly6C^{lo} and Ly6C^{lo}2 monocytes express unusually high levels of CCR2 and accumulate in the periphery (PB/Spl). MHCII^{+} monocyte population is also accumulated in the periphery.

Supplementary Tables

Table S1. Cell surface phenotype signatures for identification of distinct mature mononuclear phagocytes used in the study.

Population	Phenotype
$Ly6C^{hi}$ monocytes	$CD45^{+}Lin^{neg}CD117^{neg}CD11b^{+}CD115^{+}CX_3CR1^{+}Ly6C^{hi}F4/80^{lo/-}CD11c^{neg}I\text{-}A/I\text{-}E^{lo/-}CD43^{neg}$
$Ly6C^{lo}$ monocytes	$CD45^{+}Lin^{neg}CD117^{neg}CD11b^{+}CD115^{+}CX_3CR1^{+}Ly6C^{lo/-}F4/80^{lo/-}CD11c^{+}I\text{-}A/I\text{-}E^{lo/-}CD43^{+}$
$Ly6C^{lo}2$ monocytes	$CD45^{+}Lin^{neg}CD117^{neg}CD11b^{+}CD115^{+}CX_3CR1^{+}Ly6C^{lo/-}F4/80^{lo/-}CD11c^{neg}I\text{-}A/I\text{-}E^{lo/-}CD43^{+}$
$MHCII^{+}$ monocytes	$CD45^{+}Lin^{neg}CD117^{neg}CD11b^{+}CD115^{+}CX_3CR1^{+}Ly6C^{lo/-}F4/80^{lo/-}CD11c^{neg}I\text{-}A/I\text{-}E^{+}CD43^{neg}$
DC	$CD45^{+}Lin^{neg}CD117^{neg}CD11b^{+}CD115^{+}CX_3CR1^{+}Ly6C^{lo/-}F4/80^{lo/-}CD11c^{+}I\text{-}A/I\text{-}E^{+}CD43^{neg}$
MF	$CD45^{+}Lin^{neg}CD117^{neg}CD11b^{+}CD115^{+}CX_3CR1^{+}Ly6C^{lo/-}F4/80^{hi}$

Lin: CD3, CD45R/B220, CD19, NK1.1, Ly6G, Ter119, Ly6A/E.

DC – Dendritic cell; **MF** – Macrophage.

Table S2. Cell surface phenotype signatures for identification of distinct myeloid progenitors used in the study.

Population	Phenotype
CMP	CD45^{+}Lin^{neg}CD34^{+}CD117^{+}CD135^{+}CD11b$^{lo/neg}$CD115^{neg}CX$_3$CR1^{neg}CD16/32^{lo}Ly6C^{neg}CD11c^{neg} I-A/I-E^{neg}
CMP2	CD45^{+}Lin^{neg}CD34^{+}CD117^{+}CD135^{neg}CD11b$^{lo/neg}$CD115^{neg}CX$_3$CR1^{neg}CD16/32^{lo}Ly6C^{neg}CD11c^{neg} I-A/I-E^{neg}
GMP	CD45^{+}Lin^{neg}CD34^{+}CD117^{+}CD135^{neg}CD11b$^{lo/neg}$CD115^{neg}CX$_3$CR1^{neg}CD16/32^{hi}Ly6C^{neg}CD11c^{neg} I-A/I-E^{neg}
GP	CD45^{+}Lin^{neg}CD34^{+}CD117^{+}CD135^{neg}CD11b$^{lo/neg}$CD115^{neg}CX$_3$CR1^{neg}CD16/32^{hi}Ly6C^{+}CD11c^{neg} I-A/I-E^{neg}
MDP	CD45^{+}Lin^{neg}CD34^{+}CD117^{+}CD135^{+}CD11b$^{lo/neg}$CD115^{+}CX$_3$CR1^{+}CD16/32^{lo}Ly6C^{neg}CD11c^{neg} I-A/I-E^{neg}
cMoP	CD45^{+}Lin^{neg}CD34^{+}CD117^{+}CD135^{neg}CD11b$^{lo/neg}$CD115^{+}CX$_3$CR1^{+}CD16/32^{hi}Ly6C^{+}CD11c^{neg} I-A/I-E^{neg}
MDP2	CD45^{+}Lin^{neg}CD34^{+}CD117^{neg}CD135^{neg}CD11b$^{lo/neg}$CD115^{+}CX$_3$CR1^{+}CD16/32^{lo}Ly6C^{+}CD11c^{neg} I-A/I-E^{neg}
CDP	CD45^{+}Lin^{neg}CD34^{+}CD117^{neg}CD135^{+}CD11b$^{lo/neg}$CD115^{+}CX$_3$CR1^{+}CD16/32^{lo}Ly6C^{neg}CD11c^{neg} I-A/I-E^{neg}
pMo1	CD45^{+}Lin^{neg}CD34^{+}CD117^{neg}CD135^{neg}CD11b$^{lo/neg}$CD115^{+}CX$_3$CR1^{+}CD16/32^{hi}Ly6C^{hi}CD11c^{neg} I-A/I-E^{neg}
pMo2	CD45^{+}Lin^{neg}CD34^{neg}CD117^{neg}CD135^{neg}CD11b$^{lo/neg}$CD115^{+}CX$_3$CR1^{+}CD16/32^{hi}Ly6C^{hi}CD11c^{neg} I-A/I-E^{neg}

Lin: CD3, CD45R/B220, CD19, NK1.1, Ly6G, Ter119, Ly6A/E.

CMP – Common Myeloid Progenitor; **GMP** – Granulocyte-Monocyte Progenitor; **GP** – Granulocyte Progenitor; **MDP** – Monocyte Dendritic cell Progenitor; **cMoP** – Common Monocyte Progenitor; **CDP** – Common Dendritic cell Progenitor; **pMo** – Pre-monocyte.

Table S3. Subsets of BM cells sorted for scRNA-seq analysis.

Subset	Phenotype	Ratio in the sample
#1 ('Progenitors')	$Lin^{neg}CD115^{+}CD11b^{lo/-}CD34^{+}CD117^{+}$	1
#2 ('Pre-monocytes')	$Lin^{neg}CD115^{+}CD11b^{lo/-}CD34^{lo/-}CD117^{lo/-}Ly6C^{hi}$	1
#3 ('$Ly6C^{hi}$ monocytes')	$Lin^{neg}CD115^{+}CD11b^{+}CD34^{neg}CD117^{lo/-}Ly6C^{hi}$	1.5
#4 ('$Ly6C^{lo}$ cells')	$Lin^{neg}CD115^{+}CD117^{neg}Ly6C^{lo/-}$	2.75

Lin: CD90.2, CD3, CD45R/B220, CD19, NK1.1, Ly6G, Ter119, Ly6A/E.

Table S4. Mean frequency of converted cell subsets *in vitro*, related to Figure 7.

wt	GC-like CD177$^+$			DC-like CD319$^+$		
	Ly6C^{lo}	MoDC	MHCII$^+$	Ly6C^{lo}	MoDC	MHCII$^+$
Ctrl	4 ± 0.4	1 ± 0.1	1 ± 0.1	9 ± 1.7	23 ± 2.8	2 ± 0.3
DLL1	23 ± 0.8	4 ± 0.4	1 ± 0.1	21 ± 2.7	40 ± 3.5	1 ± 0.2
DLL4	25 ± 1.5	17 ± 2	2 ± 0.1	19 ± 2.6	49 ± 5.3	1 ± 0.2
N2$^{\Delta Cx3cr1}$	GC-like CD177$^+$			DC-like CD319$^+$		
	Ly6C^{lo}	MoDC	MHCII$^+$	Ly6C^{lo}	MoDC	MHCII$^+$
Ctrl	4 ± 0.6	1 ± 0.2	0.30 ± 0.10	9 ± 1.3	14 ± 3.3	2 ± 0.1
DLL1	7 ± 0.5	4 ± 0.8	1.44 ± 0.24	11 ± 1.7	19 ± 2.5	2 ± 0.2
DLL4	10 ± 0.3	25 ± 2.9	3.60 ± 0.25	13 ± 1.2	34 ± 2.7	2 ± 0.2

GC-like CD177$^+$ and DC-like CD319$^+$ Ly6C^{hi} monocytes were sorted from wt or *N2*$^{\Delta Cx3cr1}$ BM and cultured *in vitro* in the presence of Ctrl, DLL1, or DLL4 ligands and M-CSF (+M) and their conversion was analyzed using flow cytometry (See also **Figures 7A and 7B**). The relative frequency of Ly6C^{lo} monocytes, MHCII$^+$ monocytes, and MoDCs are shown in the table as mean±SEM. Pooled from four experiments (n=8-12)

Table S5: HTOs for sample multiplexing for scRNA-seq.

HTO	Barcode sequence
TotalSeq™-A0301 (HTO #A1)	ACCCACCAGTAAGAC
TotalSeq™-A0302 (HTO #A2)	GGTCGAGAGCATTCA
TotalSeq™-A0303 (HTO #A3)	CTTGCCGCATGTCAT
TotalSeq™-A0304 (HTO #A4)	AAAGCATTCTTCACG
TotalSeq™-A0305 (HTO #A5)	CTTTGTCTTTGTGAG
TotalSeq™-A0306 (HTO #A6)	TATGCTGCCACGGTA

Table S6: Primers for QRT-PCR.

Gene	Primer sequence
Notch2	Forward: AGTGTCAGAGGCCAGCAAGAAGAA<br>Reverse: TGATTGTCGTCCATCAGAGCACCA
Notch1	Forward: TGGAGGTCTCAGTGGCTATAA<br>Reverse: ATTCTGGCATGGGTTAGAAAGA
Hey2	Forward: TGAAGCGCCCTTGTGAGGAA<br>Reverse: TTGTAGCGTGCCCAGGGTAA
Hes1	Forward: CCGGACAAACCAAAGACGGC<br>Reverse: GGAATGCCGGGAGCTATCTTTCT
Cd74	Forward: CGCCCTAGAGAGCCAGAAAG<br>Reverse: AGGAAGTAAGCAGTGGTGGC
H2-Ab1	Forward: TGACTGCCATTACCTGTGCC<br>Reverse: GTAGCACTCGCCCATGAACT
Mertk	Forward: AGAAGGAGAGTCCAGGAAACAA<br>Reverse: CGCTCTATAATTTACGACCAGTTG
Zbtb46	Forward: AGAGAGCACATGAAGCGACA<br>Reverse: CTGGCTGCAGACATGAACAC
